# The Association between Maternal Experiences of Interpersonal Discrimination and Adverse Birth Outcomes: A Systematic Review of the Evidence

**DOI:** 10.3390/ijerph18041465

**Published:** 2021-02-04

**Authors:** Anders Larrabee Sonderlund, Antoinette Schoenthaler, Trine Thilsing

**Affiliations:** 1 Department of Public Health, University of Southern Denmark, 5230 Odense, Denmark; tthilsing@health.sdu.dk; 2 Department of Population Health, New York University, New York City, NY 10003, USA; Antoinette.Schoenthaler@nyulangone.org

**Keywords:** discrimination, birth outcomes, women’s health

## Abstract

In the present systematic review, we argue that maternal experiences of interpersonal discrimination at least partially account for the disproportionate rates of adverse birth outcomes in minority populations. Since the 1990s, research in this area has slowly, but steadily increased, shedding more light on the insidious nature of interpersonal discrimination and its toxic health effects. With the aim of bringing this topic to the fore in academic as well as clinical settings, this paper provides a state-of-the-art review of the empirical knowledge on the relationship between maternal experiences of discrimination and birth outcomes. Of 5901 articles retained in the literature search, 28 met the predefined inclusion criteria. Accounting for a range of health and behavioral factors, the vast majority of these studies support the notion that maternal experiences of interpersonal discrimination predict a range of adverse birth outcomes, including preterm birth, low birth weight, and various physiological markers of stress (allostatic load) in both mother and child pre- and postpartum. Several moderators and mediators of this relationship were also identified. These related primarily to the type (first-hand and vicarious), timing (childhood, adolescence, and adulthood), frequency, and pervasiveness of discrimination experienced, as well as to maternal mental health and coping. More research into these factors, however, is required to definitively determine their significance. We discuss these findings as they relate to the general health repercussions of interpersonal discrimination, as well as in terms of applied prenatal care and interventions. Ultimately, we argue that assessing maternal experiences of interpersonal discrimination in prenatal care may represent a considerable asset for mitigating existing majority-minority disparities in adverse birth outcomes.

## 1. Introduction

### 1.1. Background

Adverse birth outcomes represent a significant public health issue. In developed countries, over 10% (1.25 million) of all births are preterm [1,2,3,4,5,6,7], and approximately 8% (1 million) are low birth weight (LBW) [8,9,10,11]. Preterm birth (PTB) and LBW comprise two of the biggest risk factors for infant mortality and contribute to a host of lifelong consequences, including stunted growth, learning disabilities, and various chronic conditions such as obesity and diabetes [9,10]. The likelihood of adverse birth outcomes is far from equally distributed across the population, affecting minority status and stigmatized populations at a disproportionate rate. Indeed, a central and persistent risk factor—and the focus of this review—relates to the discrimination experienced by minorities. Current evidence clearly demonstrates existing and persistent disparities in birth outcomes along ethnic and racial lines. In the US, for example, Black women are significantly more likely than White women to give birth preterm (18.3% vs. 11.5%, respectively), have LBW infants (13.8% vs. 7.2%, respectively), and/or have their child die in the first year of life (1.3% vs. 0.6%, respectively) [12,13]. Similarly, ethnic minority women in the EU have higher rates of PTB and LBW infants than their majority counterparts, as do Māori populations in New Zealand, and Australian Aboriginals [8,14]. Importantly, these discrepancies in birth outcomes persist even when accounting for other known risk factors. These include, but are not limited to, quality of pre- and postnatal care, maternal comorbidity during pregnancy, parity, age, socio-economic status (SES), education, and a range of behavioral and environmental factors, such as smoking/alcohol use, poor diet, pollution, and living in deprived areas [15,16]. In other words, there is something beyond these factors that increases the risk of adverse birth outcomes disproportionately for minorities compared to the general population.

By way of explanation, the literature points to the increased burden of everyday psychosocial stress borne by minority populations. Past research particularly emphasizes the stress associated with the persistent interpersonal discrimination that minorities are uniquely subjected to on a regular basis throughout their lives [17]. This evidence centers on the chronic physiological repercussions of this type of stress and the related health implications. Specifically, allostatic load (AL), denotes the activation of the hypothalamic–pituitary–adrenal (HPA) axis in response to persistent chronic external stressors (such as e.g., discrimination) [18]. Over time, AL significantly weathers the sympathetic nervous system, increasing the individual’s risk of a range of chronic illnesses and physical complications, including cardiovascular disease, diabetes, and cancer [19,20,21]. Thus, all other things being equal, it may be the singular stress associated with the regular experience of discrimination that accounts for the increased rates of adverse birth outcomes in minority populations.

Within this context, two separate lines of research have extensively documented the positive associations between both discrimination and AL and between AL and adverse pregnancy and birth outcomes. For example, in a comprehensive meta-analysis, Pascoe and Richman [6] extracted data from 134 empirical samples and found that perceived discrimination correlated positively and strongly with psychological stress and AL. Similarly, numerous studies have found positive associations between maternal AL and risk of PTB and LBW, as well as various other pregnancy-related complications, including preeclampsia [5,19,22,23].

Integrating this evidence, emerging scientific studies have directly examined the extent to which maternal experiences of interpersonal discrimination predict adverse birth outcomes in minority populations [17,24,25,26]. Most of this research concludes in favor of a significant link. However, spanning just over 20 years and featuring multiple research designs and methodologies, it also comprises a somewhat disjointed empirical narrative. Thus, the aim of the present paper is to consolidate, review, and synthesize existing knowledge on the relationship between maternal experiences of interpersonal discrimination and adverse birth outcomes in minority populations.

### 1.2. Rationale

We identified three systematic reviews of the evidence on the link between maternal experiences of interpersonal discrimination and adverse birth outcomes. In 2011 Giurgescu et al. [13] appraised 10 relevant studies and found an overall positive relationship between interpersonal racial discrimination and likelihood of PTB and LBW. Next, in 2016, Alhusen et al. [27] retained nine quantitative studies (and four qualitative) on the effects of interpersonal racism on minority birth outcomes. Consistent with Giurgescu et al., the authors concluded that racial discrimination predicted PTB, LBW, and infant size for gestational age. However, they also found that the evidence on the matter was somewhat mixed and complicated by variable research designs across studies. Finally, Mutambudzi et al. [28] reviewed 12 studies in 2016 and argued that racial discrimination accounted for at least some of the disparity in adverse birth outcomes between Black and White American women, but also that the evidence base was small and limited. Thus, while all three reviews share the general conclusion that racial disparities in birth outcomes are related to maternal experiences of discrimination, they also highlight the relatively limited evidence base and a general lack of understanding of the mechanisms that underpin the relationship in focus. While these reviews differ slightly in terms of scope and angle, the consistency in their conclusions makes sense when acknowledging the fact that they overlap nearly perfectly in terms of the literature they assessed. Indeed, Mutambudzi et al. included two unique studies more (*N* = 12) than Giurgescu et al. (*N* = 10), and three more than Alhusen et al. (*N* = 9). For the present review, however, we have retained a total of 28 empirical studies on the topic of interest. Given the apparent growth in research in this area in the past four years, previous reviews currently appear to be considerably outdated.

In light of the confines of previous assessments of the evidence, we argue that the knowledge in this area can be significantly expanded by updating past review efforts and critically integrating new and emerging empirical findings. In particular, we will identify any relevant themes and variations in the evidence base that might have been unclear in past reviews, or that have emerged in subsequent research. In order to build a more complete, empirical model of the relationship between maternal experiences of interpersonal discrimination and adverse birth outcomes, we also aim to determine any potential pathways that might explain this relationship and highlight any gaps in knowledge that require further study. Additionally, in contrast to past efforts, we will explicitly evaluate the quality of the evidence base, using validated research quality assessment tools. Ultimately, with the present review, we hope to make two key scientific contributions:

1. To facilitate, through our review of the evidence, a greater understanding and awareness of the extent to which discrimination “gets under the skin” of minority women and their offspring, eroding their physical health and wellbeing.
2. To push this topic to the fore not only in research endeavors, but also importantly in applied health care settings. Specifically, we argue that maternal experiences of discrimination may be operationalized as a risk factor in prenatal and postpartum care and underscores the need for care approaches designed to mitigate the health repercussions associated with discrimination.

## 2. Materials and Methods

### 2.1. Protocol

This review was conducted according to the Preferred Reporting Items for Systematic Reviews and Meta-analyses (PRISMA) guidelines (see Supplementary Table S1). Full protocol details can be accessed at www.prisma-guidelines.org.

### 2.2. Literature Search Strategy

We searched a diverse range of scientific journals for literature on the association between maternal experiences of interpersonal discrimination and pregnancy outcomes in minority populations. Specifically, we executed an extensive and rigorous search of the following databases: Academic Search Premier; AMED; Global Health; SocINDEX with Full Text; CINAHL Plus with Full Text; E-Journals; MEDLINE; Psychology and Behavioral Sciences Collection; SCOPUS; Science Direct, Women’s Studies International. We also searched Web of Knowledge and Google Scholar for any references that might have been missed in the database search. Finally, we manually combed through reference lists of relevant papers and reviews for any additional articles that were not picked up in the previous two search strategies.

The predefined Boolean/phrase search terms that we employed related directly to the variables of interest: Interpersonal discrimination and pregnancy outcomes. We defined interpersonal discrimination broadly as any form of discriminating interpersonal behavior. As such, this term covered any explicit, implicit, and covert (e.g., so-called microaggressions, coded language, and behavior) person-to-person discriminatory behavior towards individuals from any minority group, including those defined by race, ethnicity, gender, sexuality, religion, nationality, and physical disability. We chose not to include studies on systemic discrimination as this type of discrimination impacts on people via pathways and processes (e.g., policies and legislation) that differ considerably from interpersonal discrimination. In terms of pregnancy outcomes, we focused on pregnancy and birth complications, including gestational age at delivery, PTB, birth weight, birth initiation (spontaneous vs. induced), and stillbirth. Other outcomes of interest included postpartum child development and health.

The exact literature search syntax was as follows: “Discrimination” and/or “prejudice” and/or “stereotype” and/or “bias” and/or “stigma” and/or “racism” and/or “sexism” and/or “ageism” AND “birth” and/or “birth outcomes” and/or “delivery outcomes” and/or “vaginal birth” and/or “labor induction” and/or “preterm birth” and/or “gestation” and/or “birth weight” and/or “stillbirth” and/or “birth complications” and/or “pregnancy complications”. Search limiters were specified to exclude non-peer-reviewed studies, newspaper articles, and non-English language text. We did not limit search results by publication date. Once the search had been executed, articles were scrutinized and retained based on the following inclusion criteria:

1. The paper reported empirical studies on the relationship between the maternal experience of interpersonal discrimination and pregnancy- and/or birth-related outcomes.
2. The paper reported quantitative results.
3. The paper was in English.
4. The full text was available.
5. The paper had undergone scientific peer review.

**Figure 1:**
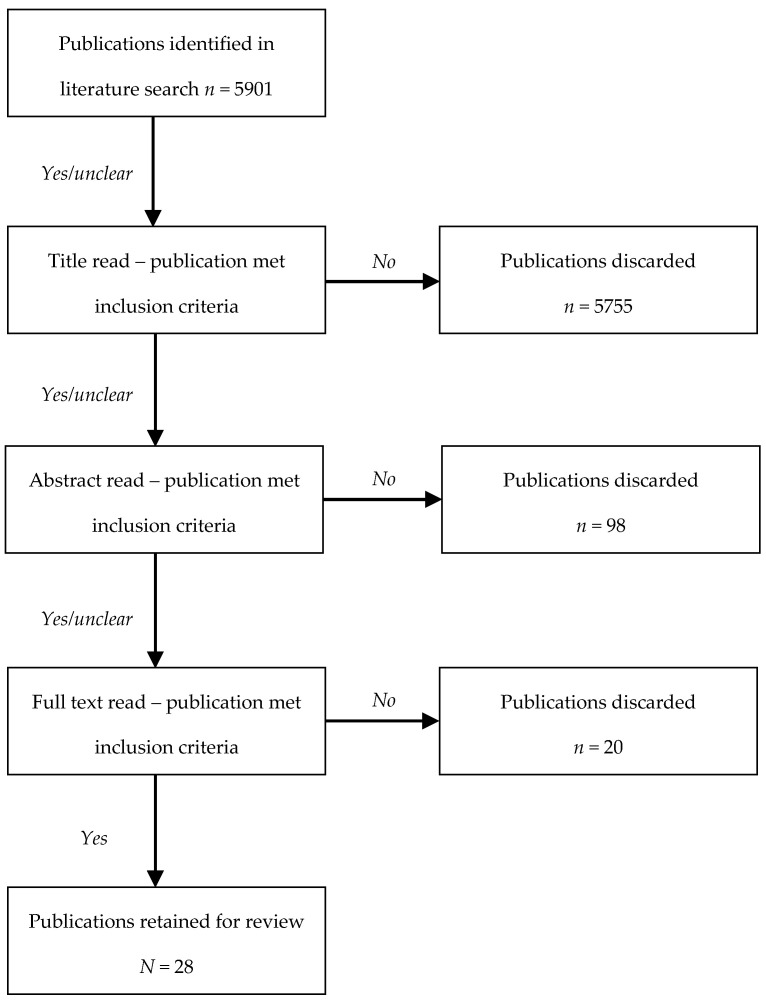
Flow chart for each step of article evaluation and retainment.

Each database hit was evaluated by the authors in three rounds against the inclusion criteria. First, papers that clearly did not relate to the topic of interest were rejected (usually based on title). Second, abstracts of papers retained in the first round were reviewed. Any articles that failed to meet the inclusion criteria were discarded. Finally, the search results that remained after the first two rounds of evaluation were downloaded and examined in full-text detail for relevance (see Figure 1). Only papers that passed through each of these three rounds were included in the present review.

### 2.3. Research Quality Appraisal

In the final round of results evaluation, we systematically appraised the methodological quality of the retained papers. To this end, we used the Quality Assessment Tool for Quantitative Studies (QATQS) [29]. The QATQS evaluates research along six dimensions, including study population selection bias, study design, confounding variables, researcher blinding, data collection methods, and participant withdrawal, and attrition. Each dimensional score for a given paper is averaged and combined into an overall assessment of the paper in terms of “weak”, “moderate”, or “strong” quality. The primary author and a qualified research assistant independently coded each paper retained for the review. Any discrepancies in the final assessment outcome for any given paper were resolved through discussion and re-examination of the paper(s) in question.

## 3. Results

### 3.1. Literature Search Results

The database search turned up a total of 5901 hits (Figure 1). Of these, 146 papers were identified that in one way or another concerned the association between discrimination and pregnancy. The vast majority of these articles were rejected based on one or more of the following reasons: The paper focused exclusively on types of discrimination other than interpersonal discrimination (e.g., structural/systemic discrimination) (*n* = 80); the paper reported on mental health issues (e.g., pre- or post-natal depression) rather than physical pregnancy- or birth-related health complications for mother or baby (*n* = 13); the paper did not cite empirical research (e.g., editorial, comment) (*n* = 6); the paper reported exclusively on qualitative results (*n* = 14); the paper provided insufficient statistical or methodological detail for assessment (*n* = 5); or a combination of some or all of these issues. Ultimately, a total of 28 papers were retained for the review. In the following sections, we will present the main findings from these studies.

### 3.2. Study Characteristics and Methodology

Of the 28 papers included in the present review, a single study was published in the 1990s, nine studies were published in the 2000s, 17 articles were published in the 2010s, and one paper was published in 2020. As such, the evidence included in this review spans four decades, though all but two studies were published between 2000 and 2019. Further, 25 articles reported on US-based studies. The remaining three papers were from Germany (*n* = 1) and New Zealand (*n* = 2). In terms of study populations, all of the articles centered on racial/ethnic minorities in White-majority societies. Most focused purely on Black women in the US (*n* = 21); however, papers also reported on Turkish immigrants in Germany (*n* = 1), ethnic minorities in New Zealand (*n* = 2), or Latina, Asian, Dominican, and/or Puerto Rican women in the US (*n* = 4). The average sample size across studies was N = 1602 (min = 39, max = 11,582, median = 480). Various research designs were employed in the retained papers, including prospective cohort (*n* = 10), cross-sectional (*n* = 7), case-control (*n* = 5), hybrid retrospective/prospective cohort (*n* = 2), retrospective cohort (*n* = 1), descriptive correlational (*n* = 1), and longitudinal designs (*n* = 2).

The main predictor and outcome measures varied somewhat across studies (see Table 1). In all of the articles, interpersonal discrimination was assessed in terms of racial or ethnic discrimination. Three studies included other bases for discrimination such as gender (*n* = 2) [30,31] and/or age, religion, physical appearance, sexual orientation, SES, nationality, and physical disability (*n* = 1) [2]. Most studies assessed discrimination with validated scales (*n* = 23), including Krieger et al.’s [32] Experiences of Discrimination Scale (*n* = 13) [3,4,30,31,33,34,35,36,37,38,39,40,41], Williams et al.’s [42] Everyday Discrimination Scale (*n* = 5) [2,43,44,45,46], the Racism and Life Experiences scale (RALES) (*n* = 3) [43,47,48], the Perceived Racism scale (*n* = 2) [34,49], and the Daily Life Experiences of Racism and Bother (DLE-B) scale (*n* = 1) [50]. Six studies either did not report which scales were implemented to measure discrimination or had devised one for the specific purposes of the given research [7,51,52,53,54,55]. Most studies operationalized discrimination in terms of frequency within a given timespan (e.g., every day, during pregnancy, past 12 months, lifetime) (*n* = 13) [2,30,31,43,45,46,47,48,49,50,52,54,55], pervasiveness in individuals’ lives (i.e., in how many different life domains has discrimination been experienced] (*n* = 7) [3,7,33,35,39,40,41], or both frequency and pervasiveness (*n* = 7) [4,26,34,36,38,44,53]. Several studies also distinguished between first-hand vs. vicarious experiences of discrimination (*n* = 5) [4,39,43,44,48], while others included measures of subjective severity of discriminatory experiences (*n* = 4) [43,47,50,52]. In terms of outcome variables, most articles reported on PTB (*n* = 14), birth weight (*n* = 14), gestational age at birth (*n* = 7), or a combination of these. PTB was defined as less than 37 weeks of gestation in all relevant studies. Studies also focused on maternal and child physiological outcomes, however, including hypertension, maternal and child cortisol secretion and stress reactivity, inflammation, and diastolic blood pressure (*n* = 4).

### 3.3. Research Methodology and Quality

The inter-rater research quality evaluations were conducted by the first author and a research assistant. Overall, independent assessments were well-aligned (see Supplementary Table S2 QATQS). Specifically, the initial round of assessments deviated on only one qualitative dimension (population selection bias in two papers, and confounding variables in one) in each of three papers (88.9% inter-rater reliability). These discrepancies were resolved through reexamination of the given papers and subsequent discussion. Ultimately, scoring each paper on each of the six QATQS dimensions, 12 papers were assessed as ‘strong’ and thus reported high-quality evidence, 13 were based on evidence of ‘moderate’ quality, and three articles were deemed to be of ‘weak’ quality (see Table 1). The current evidence base on the association between maternal experiences of discrimination and birth outcomes thus comprises research of overwhelmingly moderate-strong methodological quality.

### 3.4. Study Findings

In the following paragraphs, a brief description will be given of the main findings of each of the papers retained for this review (see Table 1 for details on study samples, research design and methodology). Unless otherwise stated, the results summarized here are adjusted for all covariates included in a given study (see Table 1). Given the general uniformity across articles in terms of outcome variables, we will present the evidence in three main sections based on explicit outcome variable. Specifically, we will first review research that focuses on the impact of maternal experiences of interpersonal discrimination on gestational age at birth (*n* = 15), before moving on to studies that look at infant birth weight (*n* = 14). Finally, we review evidence on pregnancy-related physiological outcomes (*n* = 4). While we categorize results by the outcome definitions provided in each article, we consider it pertinent to acknowledge the potential interrelationships between these outcomes. For example, low birth weight is often comprised of potentially overlapping outcomes of preterm birth and/or fetal growth restriction (i.e., small for gestational age). Thus, in spite of our categorical presentation of the evidence in terms of outcome, we implore the reader to consider these effects as potentially interconnected.

#### 3.4.1. Maternal Experiences of Interpersonal Discrimination and Infant Gestational Age at Birth

In a population-based cross-sectional study, using data from the 2004–2012 Pregnancy Risk Assessment Monitoring System, Bower et al. [51] assessed the extent to which experiences of racism accounted for racial disparities in birth outcomes in a sample of Black women (*N* = 11,582). The study sample included people from 11 US states and New York City. The authors focused on the extent to which participants had been upset by experiences of racism in the 12 months prior to giving birth. They also tested whether prenatal care moderated any such relationship. Taking into account a range of socioeconomic and health-related variables, results indicated that compared to mothers who did not report feeling upset by experienced racism, mothers who had been upset by such racism (*n* = 1645) had 29% higher odds of giving birth preterm (OR = 1.29, 95% CI, 1.04–1.59). While this relationship appeared to vary by level of prenatal care, the interaction term was statistically non-significant.

A similar cross-sectional study by Daniels et al. [44] drew data from the African-American Women’s Heart & Health Study (AAWHHS; *N* = 173) to examine the association between preterm labor and direct and vicarious racial discrimination among Black women. The study specifically concerned frequency of racism experienced in childhood, adolescence, and/or adulthood. The authors found that for each one-unit increase in reported direct racial discrimination during adolescence (measured using the Everyday Discrimination Scale) the odds of PTB increased by 48% (OR = 1.48, 95% CI, 1.00–2.19). Likewise, each one-unit increase in reported vicarious racial discrimination (i.e., witnessing discrimination perpetrated against a similar other) experienced in childhood was associated with a 45% increase in odds of preterm labor (OR = 1.45, 95% CI, 1.01–2.09). There was no relationship between adult direct or vicarious racial discrimination and PTB.

In a prospective cohort study of 1962 pregnant women in Central North Carolina between 1996 and 2000, Dole et al. [30] investigated the link between PTB and maternal stress from multiple sources, including everyday racial discrimination. Accounting for a broad range of psychosocial factors, results showed that the experience of racial discrimination was associated with a 40% increased risk of PTB. This effect was replicated in a follow-up study that revealed an 80% (RR = 1.8, 95% CI, 1.1–2.9) increased risk of PTB for Black women who had experienced high levels (as opposed to low levels) of racial discrimination. This study also indicated a 110% increased risk among Black women who had experienced gender discrimination [31].

Another prospective cohort study focused on the link between birth outcomes and frequency of lifetime experiences of racial discrimination. This research included a sample of 352 Black (*n* = 152) and White (*n* = 200) pregnant women in Birmingham, AL, Oakland, CA, Chicago, IL, and Minneapolis, MN [41]. Consistent with the aforementioned studies, results showed that Black women were more than twice as likely (21.1% vs. 10%) to give birth preterm than White women. Further, of Black women who gave birth preterm, 50% (*n* = 16) had experienced racial discrimination in at least three situations. By contrast, only 5% (*n* = 1) of White women who had given birth preterm had experienced any discrimination. Further analyses revealed that the odds for PTB among women who had experienced three or more instances of racial discrimination were 205% higher (OR = 3.05, 95% CI, 1.29–7.24) than for women who had not experienced any such discrimination.

In a case-control repeated-measures study of 277 Black women in Chicago, IL, Rankin et al. [49] found that lifetime and past-year maternal experiences of racial discrimination were positively associated with odds of PTB. In particular, women who reported high-level (as opposed to medium- or low-level) lifetime exposure to racial discrimination had 50% greater odds of giving birth preterm than women who reported little or no exposure. Similarly, women who reported high-level exposure to racism in the 12 months prior to giving birth had 150% higher odds of PTB than women reporting medium- and low-level exposures. Rankin et al. also assessed the extent to which coping style moderated the impact of racial discrimination on birth outcomes. While passive coping mechanisms (e.g., internalizing, avoiding, or ignoring the event) appeared to have no moderating effect, active coping behaviors did. Specifically, women who dealt with racial discrimination by “working harder to prove the perpetrator wrong” and/or “getting violent towards the perpetrator” had 20% (OR = 0.80, 95% CI, 0.40–1.90) and 70% (OR = 0.30, 95% CI, 0.10–1.70) lower odds, respectively, of giving birth preterm than participants who did not report these coping behaviors.

Another case-control study examined the link between PTB and different types of everyday racial discrimination (e.g., discrimination at work, as a customer, interacting with police or people in general). The sample included 422 Black mothers of preterm babies compared to 4544 Black mothers of full-term babies. Controlling for a wide range of health, demographic, and SES factors, Rosenberg et al. [53] found that women who had been treated unfairly at work (*n* = 251) or reported that people had acted afraid of them at least once a week (*n* = 50), had 30% (OR = 1.3, 95% CI, 1.1–1.6) and 40% (OR = 1.4, 95% CI, 1.0–1.9) higher odds of giving birth preterm, respectively, than those who did not report such treatment. For women with less than 12 years of education (*n* = 46), racial discrimination in housing, as a customer, and in terms of being feared by others, similarly increased the odds of PTB by 140% (OR = 2.4, 95% CI, 1.2–4.6), 240% (OR = 3.4, 95% CI, 1.5–7.7), and 100% (OR = 2.00, 95% CI, 1.0–4.1), respectively.

In a similar retrospective cohort study on the impact of racial microaggressions on birth outcomes, Slaughter-Acey et al. [50] found that the frequency and stressfulness of everyday maternal experiences of racial microaggressions predicted PTB. This research centered on a sample of Black women from Detroit, MI, (*N* = 1410). For the purposes of the study, the sample was divided into quartiles designating extent and stressfulness of experienced discrimination (first quartile = low, fourth quartile = high). Women in the 2nd to 4th discrimination quartiles (i.e., women who had experienced more and more stressful discrimination relative to women in the first quartile) had greater odds of PTB than women the first quartile. However, only differences between the 1st and 2nd quartiles were statistically significant, with women in the second quartile having 67% greater odds of PTB than women in the first quartile (OR = 1.67, 95% CI, 1.16–2.40). The authors also discovered a non-linear moderating effect of depression where women with mild-to-moderate depressive symptoms (as opposed to severe symptoms) in the second discrimination quartile had a predictive probability of PTB twice as high as their first-quartile counterparts. In other words, racial discrimination predicted PTB only in women with lower-grade depression as opposed to severe depression.

Slaughter-Acey et al.’s results resonate with those of Misra et al. [47]. They investigated the extent to which frequency of lifetime and maternal daily experiences of racial discrimination impacted on PTB in a sample of 832 Black women in Baltimore, MD. They also looked at the effect of individuals’ responses to such discrimination. Controlling for a broad range of psychosocial, demographic, and health-related risk factors, results revealed no statistically significant main effects of any measure of racial discrimination on PTB. However, women who scored above the median on lifetime exposure to racial discrimination, and who had high vs. low levels of stress or high vs. low levels of prenatal depression, had 32% (HR = 1.32, 95% CI, 0.64–3.57) and 55% (HR = 1.55, 95% CI, 0.90–2.64) increased odds of PTB, respectively. Thus, similar to Slaughter-Acey et al.’s results, Misra et al. also found a moderating effect of mental health factors, including stress and depression, on the link between racial discrimination and birth outcomes.

Finally, two studies from outside the US provide further support for the link between maternal experience of discrimination and PTB. Scholaske et al. [54] conducted a longitudinal, nation-wide panel study of pregnant non-immigrant (*n* = 2308) and Turkish immigrant women in Germany (*n* = 217). Results indicated that Turkish women who had experienced ethnic discrimination in the 12 months prior to giving birth had 319% increased odds of giving birth preterm than women who had not experienced discrimination (11.84%, χ^2^(1, 109) = 8.18, *p* < 0.01, OR = 4.19). Similarly, in a longitudinal cohort study from New Zealand, Thayer et al. [7] found significant links between pervasiveness of maternal experiences of discrimination and PTB in a sample of pregnant Māori, Pacific, and Asian women (N = 1653). Indeed, shorter gestation period was evident for Māori women who reported ethnically motivated physical attacks (*β* = −1.06 week, 95% CI, −1.8 week, −0.3 week) or unfair treatment in the workplace (*β* = −0.95 week, 95% CI, −1.6 week, −0.3 week), the criminal justice system (*β* = −0.55 week, 95% CI, −1.1 week, 0.02 week), or in banking (*β* = −0.73 week, 95% CI, −1.4 week, −0.02 week).

In addition to the 11 articles described above that all indicate a negative impact of maternal experiences of interpersonal discrimination on PTB and/or gestational age at birth, another four studies report null effects. Specifically, Gillespie and Anderson [36] conducted a prospective cohort study of 96 Black women in Ohio, US. They assessed the extent to which the frequency and pervasiveness of interpersonal discrimination in individuals’ lives predicted gestational age at birth and various physiological risk factors of PTB (see Table 1). The authors found no main effects of maternal experiences of discrimination on gestational age, and their results for physiological variables were mixed. Specifically, they found an interaction effect in the expected direction between discrimination and coping mechanisms on cortisol levels during pregnancy. Among women who tended to internalize experiences of discrimination (*n* = 32), those who experienced medium levels of discrimination exhibited higher cortisol levels (a known risk factor for PTB) during pregnancy than those who experienced low levels of discrimination (*b* = 0.68, *p* = 0.001). Overall, however, greater cortisol levels predicted earlier birth only for mothers who reported low levels of discrimination. Further, results also indicated a protective effect of discrimination on leukocyte glucocorticoid sensitivity (another risk factor for PTB).

Comparable results were generated in a study by Giurgescu et al. [37] on the link between frequency and pervasiveness of lifetime maternal experiences of racial discrimination and PTB. In a sample of Black women from Chicago, US, the authors found a significant positive association between experienced discrimination and psychological distress (*β* = 0.52, *p* < 0.01), but not PTB (*p* > 0.05). Similar to the study by Gillespie and Anderson, however, these results were based on an exceedingly small sample (*N* = 72) who reported generally low levels of discrimination.

In another two studies that collected data from much larger samples, Grobman et al. [38] (*N* = 9470) and Mendez et al. [52] (N = 3462) found no statistically significant association between PTB and frequency or pervasiveness of maternal experiences of lifetime or everyday discrimination. In particular, the former study found that while Black women had greater odds of giving birth preterm (OR = 1.6, 95% CI, 1.32–1.93) than White women, this relationship was not accounted for by discrimination. Similarly, Mendez et al. found that Black women were nearly twice as likely (14.9%) as White (7.7%) or Hispanic (8.3%) women to give birth preterm, but this disparity in PTB was not explained by interpersonal discrimination. Both Mendez et al. and Grobman et al. cited demographically uneven sample sizes and small subgroup sizes as potential explanations as to why their analyses, in contrast to the rest of the relevant evidence base, failed to detect a statistically significant contribution of interpersonal discrimination to PTB.

#### 3.4.2. Maternal Experiences of Interpersonal Discrimination and Infant Birth Weight

Two case-control studies conducted by Collins et al. in 2000 [43] and 2004 [34] focused on samples of Black women who gave birth to healthy-weight (>2500 g) children vs. very-low-birth-weight (VLBW; <1500 g) children. The former study centered on 85 Black women in Chicago, IL, 25 of whom had VLBW children, while the rest had normal-weight children. In the fully adjusted model, results indicated that the odds of infant VLBW were marginally greater for women who had been exposed to racial discrimination during pregnancy (OR = 3.3, 95% CI, 0.9–11.3). These odds increased for women who had two or more of the following risk factors: high parity, poor or no prenatal care, inadequate social support, or tobacco, alcohol, or illicit drug use (OR = 4.4, 95% CI, 1.1–18). Indeed, with the exception of education, every risk factor included in the analyses (see Table 1) appeared to interact with maternal experience of racial discrimination to increase the odds of giving birth to VLBW children. However, possibly due to the exceedingly small cell sizes, these increases were marginally significant at best.

In Collins et al.’s second study [34], the sample was bigger, but identical in characteristics, focusing on Black mothers of VLBW children (*n* = 104) vs. Black mothers of normal-weight children, (*n* = 208). The authors found that the experience of lifetime discrimination in at least one of five life domains (work, getting a job, school, medical care, and receiving service) (*n* = 58) increased the odds of giving birth to VLBW children by 90% (OR = 1.9, 95% CI, 1.2–3.1). Being the target of discrimination in at least two domains, increased the odds to 110% (OR = 2.1, 95% CI, 1.2–3.8), and having these experiences in three or more life domains, increased the odds further to 220% (OR = 3.2, 95% CI, 1.5–6.6). This pattern suggests a dose-response relationship between pervasiveness of lifetime racial discrimination and risk of infant VLBW.

Complementing the findings of Collins et al., Lespinasse et al. [40] also looked at the effect of pervasiveness of maternal lifetime experiences of racial discrimination on infant birth weight. In a case-control study of 312 mothers (mothers of VLBW infants <1500 g *n* = 104 vs. mothers of normal-weight infants ≥2500 g *n* = 208) in Chicago, IL, the authors found that experiencing racial discrimination in one or more life domains increased the odds of VLBW by 90% (OR = 1.9, 95% CI, 1.2–3.0). For women who had experienced racial discrimination in three or more life domains, odds of VLBW increased by 170% (OR = 2.7, CI 95% 1.3–5.4).

Dixon et al. [3] conducted a similar prospective cohort study on the pervasiveness of maternal racial discrimination and infant-toddler birth weight in a sample of Black, Hispanic, and Asian women (N = 539) in MA, USA. They found negative associations between maternal lifetime pervasiveness of racial discrimination and both birth and child weight. Specifically, compared to mothers who had not experienced any racial discrimination, mothers who had experienced racial discrimination in three or more life domains had children of lower weight at birth (z-score *β* = −0.25, 95% CI, −0.45 to −0.04), at six months of age (z-score *β* = −0.34, 95% CI, −0.65 to −0.03), and at three years of age (z-score *β* = −0.33, 95% CI, −0.66 to 0.00). The offspring of mothers who reported racial discrimination in 1–2 life domains were (though non-significantly) in between the two other cohorts (discrimination in 0 vs. >3 life domains) in terms of weight, suggesting a dose-response relationship.

Next, in a cross-sectional study of Black (*n* = 407) and White (*n* = 222) mothers in Saginaw County, MI, Carty et al. [43] investigated the link between frequency of interpersonal racial discrimination and child birth weight. The authors operationalized discrimination in terms of sheer frequency of experiences in the past year, perceived group racism (i.e., vicarious racism), racism-related stress, and individual emotional reaction to experienced interpersonal racism. Results indicated that only emotional reactions to racial discrimination was associated with child birth weight. Specifically, the more extreme the individual’s emotional response, the greater the odds of giving birth to a child of LBW (<2500 g) (OR = 1.24, 95% CI, 0.93–1.48). Adjusting for race and educational level, this association attenuated and was only marginally significant (OR = 1.17, 95% CI, 0.93–1.48).

Further, while the studies by Collins et al., Lespinasse et al., and Dixon et al. examined frequency and/or pervasiveness of racial discrimination in various life domains, Dominguez et al. [4] focused on the effects of discrimination experienced in different life stages (childhood, adolescence, adulthood, and lifetime). As in Carty et al., this study also operationalized racial discrimination in terms of direct and vicarious experiences. The sample comprised 124 pregnant women (Black *n* = 51, White *n* = 73) in Los Angeles, CA. Accounting for a wide range of SES, demographic, and health factors, the authors found that for each one-unit increase in overall maternal experience of lifetime racial discrimination infant birth weight decreased by 39.59 g. Breaking this association down, the authors found that racial discrimination experienced vicariously in childhood was the main driver of this effect. Indeed, for each one-unit increase in maternal vicarious childhood experiences of discrimination, infant birth weight decreased by 167.85 g (*β* = −0.25, *p* < 0.01), suggesting a dose-response relationship.

Similar to Carty et al. and Dominguez et al., Hilmert et al. [39] also investigated the extent to which the pervasiveness of direct and vicarious racial discrimination experienced in childhood and/or adulthood impacted on infant birth weight. This prospective cohort study included a sample of Black pregnant women (N = 39) in Los Angeles, CA. Total maternal racial discrimination (direct and vicarious combined) emerged as a statistically marginal predictor of birth weight (*β* = −0.27, *p* < 0.10), while direct racial discrimination experienced in adulthood accounted for a significant amount of variance in birth weight (*β* = −0.26, *p* < 0.05). Further, maternal vicarious and direct childhood experiences of racial discrimination interacted with change in prenatal diastolic blood pressure (DBP) to impact negatively on birth weight (*β* = −0.25, Δ*R*^2^ = 0.04, *p* < 0.05; *β* = −0.22, Δ*R*^2^ = 0.03, *p* = 0.10, respectively). Specifically, for women who had vicariously experienced racial discrimination in approximately two childhood domains, infant birth weight decreased by 19.98 g for every 1 mmHg increase in maternal DBP (B = −160.65, *p* < 0.05). This association was not evident in women who had experienced no such discrimination.

Extending the line of research into the impact of direct and vicarious maternal discrimination, Slaughter-Acey et al. [48] conducted a hybrid retrospective/prospective cohort study of 778 Black women in Baltimore, MD. The authors operationalized racism in terms of frequency of experienced direct racism, group-based (vicarious) racism, and overall racism (direct and vicarious combined). Results indicated an interaction between maternal age and frequency of both overall and vicarious racism. For women over the age of 25 years (*n* = 257), each one-unit increase in overall racism was associated with 45% greater odds of low infant birth weight (OR = 1.45, 95% CI, 1.02–2.08). Disentangling this effect, the authors found that the odds for low infant birth weight increased 184% for each one-unit increase in *vicariously* experienced racism in women over 25 years old (OR = 2.84, 95% CI, 1.10–7.32). A similar, but non-significant trend in direction was evident for the link between infant birth weight and *direct* racism in women over 25 years old. These associations did not emerge in the results for women under 25 years old.

Next, Earnshaw et al. [45] conducted a prospective cohort study into the association between frequency of maternal experiences of everyday discrimination and infant birth weight. They focused on a sample of Black (*n* = 158) and Latina (*n* = 262) women in New York City, NY. Consistent with the previously cited research, results indicated an inverse association between frequency of maternal everyday discrimination and infant birth weight (OR = 2.78, *p* < 0.05). Interestingly, the authors also found that this association was mediated by increased depressive symptoms during pregnancy (*β* = −0.04, *p* < 0.01). In raw numbers, this translates into an infant birth weight decrease of 49 g for every one-point increase in maternal everyday discrimination. While maternal prenatal stress also correlated with maternal experiences of discrimination (*β* = 0.27, *p* < 0.01), this variable did not predict or mediate birth outcome.

In a similar prospective cohort study that also took into account antenatal depression, Mustillo et al. [41] looked at pervasiveness of racial discrimination and birth weight in a sample of pregnant women in Chicago, IL, Oakland, CA, and Minneapolis, MN, (N = 352). The odds of giving birth to low-birth-weight infants was 398% (OR = 4.98, 95% CI, 1.43–17.39) greater for women who had experienced racial discrimination in >2 life domains compared to women who had experienced none. In contrast to Earnshaw et al., analyses failed to detect a statistically significant mediating effect of maternal depressive symptoms.

The only article that reported on maternal discrimination and infant birth weight and was conducted outside of the US, was the aforementioned longitudinal cohort study by Thayer et al. [7]. Complementing their findings on discrimination and gestational age at birth described in the previous section, their results also indicated a link between pervasiveness of maternal experiences of discrimination and infant birth weight. Indeed, Māori women who experienced discrimination at work or in acquiring housing had children with lower birth weight than those who had not experienced such discrimination (*β* = −243 g, 95% CI, −425 g, −60.2 g; (*β* = −146 g, 95% CI, −286 g, −6 g, respectively). Somewhat counter-intuitively, Asian women who had experienced discrimination in housing (vs. Asian women who had not experienced such discrimination) were more likely to have children of higher birth weight (*β* = 188 g, 95% CI, 7 g, 369 g).

Finally, of the articles that included infant birth weight as an outcome variable, three reported only marginal or no effect of experienced discrimination. Specifically, in a prospective cohort study of 108 Black women in Northern CA, Daiely [2] found no link between infant birth weight and maternal experience of general discrimination. Results did indicate that maternal experience of religious discrimination was negatively associated with infant birth weight (*t* = 2.39, *p* = 0.02), though given the exceedingly small cell size (only *n* = 10 women reported religious discrimination), this outcome may at best be suggestive rather than actually statistically meaningful. Similarly, while Grobman et al. [38] found that Black women had 120% higher odds than White women of giving birth to low-weight infants (OR = 2.2, 95% CI, 1.86–2.62), neither frequency nor pervasiveness of maternal discrimination accounted for this difference. The authors indicated that in spite of the overall large sample, individual cell sizes were relatively small and might have accounted for the lack of statistically significant results. Finally, in their cross-sectional study of 1150 pregnant minority and White women in Chicago, IL, and New York City, NY, Shiono et al. [55] failed to detect any statistically significant impact of frequency of maternal experience of racial discrimination on infant birth weight. Various methodological issues were suggested as potential causes for these non-significant results, including most prominently study sample diversity in terms of cultural background and English language ability/comprehension, as well as questionable accuracy of survey translations.

#### 3.4.3. Maternal Experiences of Interpersonal Discrimination and Physiological Pregnancy Outcomes

While other articles retained for this review tested physiological variables as mediators of the relationship between maternal experience of discrimination and birth outcomes (including Grobman et al., Gillespie et al., and Hilmert et al.), two studies focused explicitly on pregnancy- and/or birth-related physiological outcome variables. Christian et al. [33] examined Epstein–Barr virus (EBV) reactivation as a proxy for cellular immune competence during pregnancy and postpartum. Specifically, they focused on increased levels of EBV virus capsid antigen immunoglobulin G (VCA IgG)—a known marker of chronic stress and AL, and risk factor for adverse birth outcomes. The authors focused on a sample of pregnant women (Black *n* = 38, White *n* = 18) in Ohio, US, and measured lifetime pervasiveness of experienced maternal discrimination. Results showed that Black women exhibited substantially higher levels of IgG antibody titers than White women during each trimester and postpartum. Further, women who reported high discrimination showed higher EBV VCA IgG antibody titers than women who reported low discrimination in the first (*p* = 0.03) and second trimesters (*p* = 0.04) as well as postpartum (*p* = 0.06). The authors note that the small cell sizes represent a limitation, however, and the results should be interpreted cautiously.

In a similar study, Thayer et al. [46] assessed the extent to which frequency of experienced discrimination was associated with pregnant women’s morning and evening cortisol levels during pregnancy as well as infant cortisol levels at postpartum vaccination (as a proxy for stress reactivity). The study sample comprised an ethnically diverse group of 64 women in Auckland, New Zealand. Results indicated that women who had experienced ethnic discrimination (*n* = 22) exhibited higher levels of evening cortisol levels (*m* = 1.25 ng/mL) than women who had experienced low levels of discrimination (*m* = 0.9 ng/mL, *p* < 0.01) or none at all (*m* = 0.8 ng/mL, *p* < 0.001). Further, the infants of women who had experienced discrimination (*n* = 19) vs. those of women who had not experienced discrimination (*n* = 41), exhibited greater cortisol responses to vaccination (*β* = 6.43, SE = 2.60, *t* = 2.47, *p* < 0.05).

#### 3.4.4. Preliminary Process Model

**Figure 2:**
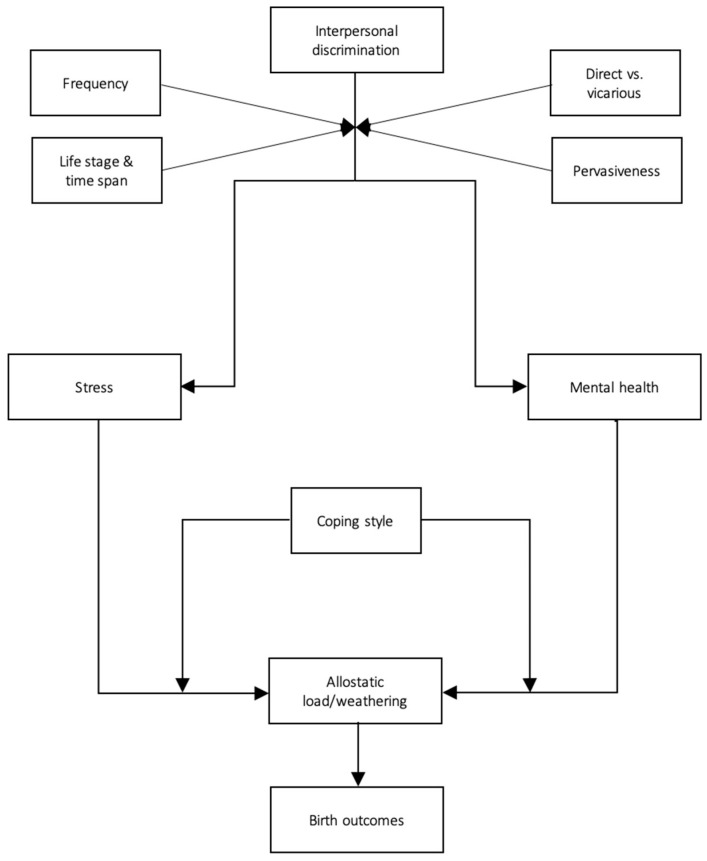
A preliminary, evidence-based model of the psychophysiological impact of interpersonal discrimination on birth outcomes.

To sum up our review of the evidence, we have created a preliminary process model for the relationship between interpersonal discrimination and adverse birth outcomes (Figure 2). While the pathways that we have defined here are somewhat tenuous given the general scarcity of studies on each specific mechanism, they are nonetheless consistent with the broader literature on the physical health repercussions of interpersonal discrimination [56,57,58].

## 4. Discussion

### 4.1. Main Findings

The results reported in the reviewed papers overwhelmingly support the conclusion that maternal experiences of interpersonal discrimination are positively associated with increased risk of adverse pregnancy outcomes. Controlling for an extensive range of covariates, 24 out of 28 articles presented empirical evidence that indicate strong links between interpersonal discrimination and infant birth weight, PTB, gestational age at birth, and both maternal and infant physiological stress reactivity. Of the remaining four studies, three reported inverse, albeit statistically non-significant connections between the variables of interest [37,38,52], and one excluded non-significant statistics from their results, preventing assessment of directionality [55]. Thus, taken together, these results resonate strongly with the extant literature on the health effects of experienced discrimination in minority populations, emphasizing the very real and physical toll of the stress caused by interpersonal discrimination [58,59,60,61,62,63,64]. This research may also have implications for prenatal and postpartum care in minority populations—for example, with regard to the potential of screening for maternal experiences of discrimination as a significant risk factor for adverse birth outcomes.

While the overall association between maternal experiences of discrimination and adverse birth outcomes is rather clear, there are nonetheless a number of notable themes and variations that emerge from the reviewed studies. These include principally the type of discrimination (e.g., frequency vs. pervasiveness, lifetime vs. past year vs. everyday experiences, vicarious vs. direct), and the recurring moderating or mediating roles of stress and depression.

In virtually all of the studies, the frequency and/or pervasiveness of interpersonal discrimination in individuals’ lives comprised the main gauge of discrimination. However, across studies, the time spans tapped for discriminatory experiences varied. Most studies (*n* = 16) measured *lifetime* experiences of discrimination, while others focused on *past-year* discrimination (*n* = 10) or *everyday* discrimination (*n* = 3). These different conceptualizations of discrimination fluctuated markedly in predictive reliability. Specifically, of the 16 studies that included measures of lifetime discrimination, all but two [37,38] found a significant association between this construct and birth outcomes. Three of the six studies that focused exclusively on past-year experiences of discrimination, however, found either no significant relationship at all [55] or generated mixed results [43,50]. Finally, four studies employed both lifetime and short-term measures of discrimination, affording a more direct comparison. In two of these studies, lifetime, but not past-year discrimination predicted birth outcomes [7,34]. The other two studies reported significant associations between both discrimination measures and birth outcomes [48,49]. Taken together, these findings indicate relatively clearly that in terms of adverse birth outcomes, lifetime experiences of discrimination are the most consequential.

Four studies also included extra measures of vicarious experiences of discrimination across the lifespan and demonstrated that discrimination need not be experienced first-hand to impact on birth outcomes. Specifically, Daniels et al. [44], Dominguez et al. [4], and Hilmert et al. [39] all found that vicarious maternal childhood experiences of racism, perpetrated against close ingroup members (e.g., family or friends), were associated with PTB and decreased birth weight. Similarly, Slaughter-Acey et al. [48] found that experiences of vicarious discrimination in women over 25 years of age were negatively associated with infant weight for gestational age. Theoretically, this effect may be due to the chronic situational vigilance (and stress) that might result from the awareness that if a relative or friend can be discriminated against, so too can the individual themselves be a target of discrimination. In other words, “merely” witnessing a close, similar other being discriminated against may have severe stress-related health implications later in life. This line of reasoning resonates well with other studies that show that stereotype threat and/or worry about becoming a target of discrimination, has comparable consequences for birth outcomes as those associated with experiences of actual discrimination [65,66]. As measures of vicarious experiences of discrimination typically, if not exclusively, relate to the experiences of “close others”; however, it is unclear whether this effect extends to more distal members of the individual’s perceived ingroup as well.

A total of 14 articles included mental health covariates of stress and/or depression in their analyses. Of these, six studies found that stress and/or depression mediated or moderated the relationship between maternal experiences of discrimination and birth outcomes [33,36,45,46,47,50]. Specifically, Slaughter-Acey et al. [50] found that depression (but not stress) moderated the effects of discrimination on birth outcomes, whereas in Misra et al. [47] both of these variables were significant moderators. Earnshaw et al. [45], however, found that depression (but not stress) mediated the effects. Further, Christian et al. [33], Gillespie et al. [36], and Thayer et al. [46]. were largely unanimous in their conclusion that maternal experiences of discrimination likely caused increases in maternal and infant stress and allostatic load markers (cortisol, cellular immune response, glucocorticoid sensitivity)—a known risk factor for adverse birth outcomes and general health. Taken together then, these results suggest that not only may depression and stress make birth outcomes more vulnerable to the impact of discrimination, but it might also be the case that discrimination gives rise to stress and depression, which in turn cause adverse birth outcomes and maternal and infant health issues. In other words, these factors may moderate *and/or* mediate the effects of maternal experiences of discrimination on birth outcomes and infant health. More research, however, is required to disentangle these relationships and definitively determine how and when stress and depression factor into this association.

The remaining eight studies that included measures of depression and/or stress found no significant explanatory power associated with any of these variables [3,4,30,37,38,43,52,55]. It should be noted, however, that all but two of these studies focused on recent symptoms of maternal depression and/or stress (typically past month or prenatal). By contrast, the six studies in which stress and/or depression *were* found to be significant predictors, used more general, life-time measures to gauge these emotions. This suggests that the mediating/moderating role of stress and depression in this context may be better captured by tapping lifetime chronic and pervasive stress and/or depression rather than confining measurement of these variables to more specific and shorter-term bouts (e.g., prenatal and postpartum). This resonates well with the findings noted above that it is *lifetime* exposure to discrimination, as opposed to more recent shorter-term exposures, which appear to impact on birth outcomes. These conclusions also dovetail nicely with the broader literature on the weathering effect of discrimination. Here, it is, namely, the cumulative build-up of discrimination-related stress and allostatic load that over time wears and tears on the body, slowly causing it to break down [56,62].

### 4.2. Strengths and Limitations

There are several strengths and limitations to the reviewed evidence base. As noted in Section 3, the quality of the reviewed studies is predominantly high, with 12 articles based on strong research methodologies, and another 13 on moderate-quality methods. These ratings were mainly due to the rigorous research designs employed (e.g., cohort studies, longitudinal designs, etc., see Table 1), the large and representative study populations, and the extensive effort to control for a broad range of covariates. Indeed, the longitudinal cohort studies by Thayer et al. [7] and Dixon et al. [3] as well as the case-control study by Rosenberg et al. [53], stand out as particularly noteworthy examples of the overall high research quality. Further, our quality assessment contrasts with those of previous reviews, which have pointed to methodological issues and limitations in the evidence base. As noted in the introduction, however, these reviews were based on a small pool of studies, less than half as big (max *N* = 12) as the present review (*N* = 28). Further, all of the studies published since these previous reviews were conducted have been of moderate or high quality (see Table 1). In other words, the evidence base appears to have not only expanded, but also qualitatively improved.

Another strength relates to the conceptualization of interpersonal discrimination in the reviewed studies. Past research has emphasized the tendency in discrimination research to define this behavior in somewhat simplistic, unidimensional terms—often as a frequency within a certain timeframe [32,58]. However, of the reviewed studies, all but two [30,31] used multidimensional scales of discrimination that tapped frequency, pervasiveness across individuals’ life domains, stressfulness, etc. This lends considerable credence to the results, as these measures are likely to have captured relatively richer, more accurate data on the nature and extent of experienced discrimination than studies focusing on more narrow definitions.

Finally, from the perspective of the broader field of discrimination, stress, and health, the rather objective dependent variable of birth outcomes and/or maternal and infant physiological stress reactivity is a clear strength. Most other studies in the area rely on self-reported health or stress—measures that are prone to bias and error given their subjective nature. The reviewed studies, however, circumvent this issue by focusing on tangible, corporeal endpoints (birth weight, PTB, and allostatic load) associated with discrimination-related stress. In conjunction with past evidence on these types of physical outcomes (such as, e.g., the literature on discrimination and hypertension), this research thus helps solidify more objective insight into the harmful biological consequences of being subjected to interpersonal discriminatory behavior.

There are also a few limitations to the evidence base that need to be noted. First and foremost, with the exception of two studies from New Zealand and one from Germany, all of the research retained for this review is from the US. Further, the vast majority of these articles focus on discrimination against Black Americans, with only a handful including other ethnicities as well. Based on the current evidence then, conclusions about the present subject matter extends nearly exclusively to Black women in the US. In other words, given the unique history of subjugation and discrimination against Black Americans, the evidence base may not generalize in the exact same way to other minority populations. Of additional relevance in this context is the fact that the for-profit model of the US health care system poses significant challenges in terms of equal access compared to other, government-funded models such as those implemented in Germany and New Zealand. Further, determining whether other bases for discrimination—be it gender, age, mental health, disability, sexuality, etc.—have comparable effects on birth outcomes is somewhat tricky, and ultimately requires further research.

Another shortcoming relates to the measure of discriminatory experiences employed in the reviewed studies. While we listed the multidimensional operationalization of discrimination as a strength above, this is mostly by comparison to existing research. That is, beyond the effects of frequency and pervasiveness, other elements of discrimination appeared largely untapped in the research or lacked in detail. For example, more knowledge is required on the extent to which vicarious experiences of discrimination are moderated by social proximity of the target. That is, are the harmful effects of vicarious experiences of discrimination dependent on the extent to which the perceiver is personally connected to the target (e.g., a “close other” vs. an acquaintance vs. an ingroup member with no personal connection to the perceiver)? This may be a pertinent avenue for future research—particularly given the increasing number of cases of racial and gender-based discrimination that make it into the public eye in the US, the EU, and elsewhere.

Further, the most common discrimination measure across studies was the Krieger Everyday Discrimination Scale (*n* = 13). While this scale has high validity and reliability scores [32], the items focus mainly on overt, active discrimination, priming the individual to recall whether they “have […] ever been prevented from doing something, been hassled, or made to feel inferior”. As such, without explicitly asking about discrimination of a more subtle, passive, or covert nature (e.g., coded language, micro-aggressions, and social invisibility), these types of experiences may not have been captured in many of the studies included in this review. Recent research, however, indicates that this type of discrimination is pervasive and harmful [67,68,69,70].

As is the case with most systematic reviews, the evidence base in focus is potentially limited by positive findings publication bias [71]. That is, any number of studies may have been conducted on this topic but rejected for publication due to statistically non-significant results.

Lastly, given the nature of the relationship between discrimination and birth outcomes, the evidence base consists exclusively of correlational data. Thus, definitive conclusions about cause and effect cannot be drawn. That said, the detailed cohort study methodology employed in most of the retained studies may be the next-best thing.

### 4.3. Theoretical, Social, and Clinical Implications

All strengths and limitations considered, the evidence synthesized in this review may have several meaningful implications. From a socio-theoretical perspective, the overarching theme that maternal experiences of interpersonal discrimination have serious and independent consequences for birth outcomes, lends further credence to the weathering effect hypothesis. While this theory has already received considerable support [72,73,74], this review indicates that discrimination not only erodes the health and wellbeing of the immediate target but may also transcend generation and cause physical harm to her unborn child. Extending the plethora of research on the mental health repercussions of interpersonal discrimination, this article thus highlights the very toxic *biological* impact associated with the perpetration of this type of behavior [58]. The growing evidence base on the exceedingly pernicious and visceral effect of interpersonal discrimination may serve to further illustrate the crucial importance of addressing this issue in a comprehensive and preventive manner. This may include, but is not limited to, the consistent prioritization of extensive schooling and critical dialogue about discrimination in core curricula at all levels of education. Similarly, public awareness campaigns and training interventions in particular settings (e.g., health care, education, and the workplace) have also shown some success [75,76]. These types of approaches might focus on illuminating the nature, mechanisms, and the implicit as well as the overt enactment of both interpersonal and institutional discrimination. The roots of discrimination run deep in most Western cultures, however, and any effective and lasting remedy will also require pervasive and meaningful bottom-up policies and legislation that target structural prejudice and discrimination and promote social and economic equity in all parts of society [76].

In addition, and from a clinical, and more reactive standpoint, the cumulation of evidence on this matter may inform various aspects of maternal health care. Incorporating measures of maternal experiences of discrimination in maternal health-risk algorithms, for instance, may improve preconception and/or prenatal care. There is also promising evidence (non-peer-reviewed) of the effectiveness of prenatal care approaches in the US that take a more patient-centered approach than most hospital protocols. These programs are based on providing extensive social, emotional, and instrumental support during pregnancy as a protective buffer against psychosocial stressors such as discrimination. Some of these have produced impressive preliminary results. Of particular note, a comprehensive evaluation of the *JJ Way*—an independent maternity care model implemented in Orange County, Florida—compared birth outcomes for their patients with those of women in prenatal care at local hospitals. They found that the JJ Way care model significantly decreased racial disparities in terms of low-birth-weight babies, and altogether eliminated the gap for preterm birth outcomes [77]. The results were attributed primarily to the stress-protective effects of the expansive interpersonal and structural support system that formed the bedrock of this prenatal care model. The key components of the *JJ Way* center specifically on building the social capital of the women in their care by creating a safe pre- and postnatal environment based on interpersonal respect, support, education, and empowerment. These types of approaches fit well with the broader literature on the buffering effect of social support and connectedness against stress, allostatic load, and disease [78].

## 5. Conclusions

Discrimination is an omnipresent, persistent, and insidious societal issue. This review contributes to the growing body of literature on the link between discrimination and health. Specifically, it emphasizes the fact that maternal experiences of interpersonal discrimination have grave physical implications that may extend beyond women’s individual health to negatively affect their pregnancy and offspring. As illustrated in Figure 2, this effect appears to occur via chronic mental strain (stress, depression) and associated allostatic load. However, given the relative scarcity of research on the specific mediating and moderating factors, the illustration serves primarily as a jumping-off point for future research than a definitive exposé of the mechanisms of the relationship. Future study should delve deeper into these processes to more definitively confirm their place and role in the relationship between interpersonal discrimination and adverse birth outcomes. Specifically, the maternal health repercussions of vicarious discrimination and lifelong vs. prenatal experiences of discrimination require further study. So too, does the role of implicit vs. explicit discrimination, as well as childhood vs. adult experiences. Crystallizing the pathways that underpin the relationship in focus may help inform and specify interventions, risk algorithms, and clinical care, and ultimately decrease the ethnic and racial disparities in adverse birth outcomes.

## Figures and Tables

**Table 1:** Design, samples, and methodology for research studies included in present review.

Author	Location	Population (N)	Research Design	Discrimination Measure (Scale Name)	Covariates	Outcome Variables	Findings	Research Quality
Bower et al. (2018) [51]	12 states, USA	Non-Hispanic Black mothers (*N* = 11,582).	Cross-sectional	Experienced discrimination in past year (single-item, binary measure).	Adequacy of prenatal care Age Education Marital status Maternal smoking Pre-pregnancy BMI Pre-pregnancy insurance status (proxy SES) Regional differences.	Preterm birth (<37 w)	Overall, 14.2% (95% CI, 13.3–15.2) of participants had experienced racism in the past year. Controlling for all covariates, women who had experienced racism in the past year were 1.29 (95% CI, 1.04–1.59) more likely than women who had not experienced racism to give birth preterm. Moderation analyses indicated that experience of racism was positively associated with odds of preterm birth for women who received intermediate (AOR, 2.03, 95% CI, 1.04–3.97) or adequate prenatal care (AOR, 1.57, 95% CI, 0.95–2.59). There was no association for inadequate or adequate-plus prenatal care.	Moderate
Carty et al. (2011) [43]	Saginaw County, MI, USA	Mothers (N = 629, Black *n* = 407, White *n* = 222).	Cross-sectional	Frequency of experiences of past-year racism (Everyday Discrimination Scale) Perceived group-impact racism (Racism and Life Experiences Scale) Racism-related stress (Racism and Life Experiences scale) Emotional reactions to racism (Racism-related Experiences Scale).	Age Parity Education Self-identified race Beliefs and experiences of racism Self-reported physical/mental health Smoking Pregnancy and birth experiences.	Birth weight	Frequency of racial discrimination was positively associated with likelihood of smoking and negatively correlated with mental and physical health. Emotional responses to racism was the only racism measure that predicted pregnancy or birth outcome. Experiencing racism that elicited emotional reactions increased the odds of low birth weight by 24% (OR = 1.24, 95% CI, 0.93–1.48). This effect attenuated in the fully-adjusted model, rendering the statistical effect marginally significant.	Moderate
Christian et al. (2012) [33]	OH, USA	Pregnant women (N = 56, Black *n* = 38, White *n* = 18).	Longitudinal	Pervasiveness of lifetime experiences of discrimination (Experiences of Discrimination Scale).	Age Race/ethnicity Marital status Education Income Gravidity Parity Pre-pregnancy BMI Depression Stress (general) Stress (pregnancy-related) Anxiety Smoking Exercise Prenatal vitamin use.	Epstein-Barr virus reactivation (IgG antibody titers) during pregnancy and post-partum.	Black women exhibited substantially higher levels of IgG antibody titers than White women during each trimester and postpartum: 1st trimester, m = 3.13 (95% CI, 3.02–3.26) vs. m = 2.62 (95% CI, 2.45–2.80), respectively. 2nd trimester, m = 3.13 (95% CI, 3.01–3.25) vs. m = 2.62 (95% CI, 2.47–2.81), respectively. 3rd trimester, m = 3.09 (95% CI, 2.97–3.20) vs. m = 2.64 (2.47–2.81), respectively. Postpartum, m = 3.14 (95% CI, 3.02–3.26) vs. m = 2.66 (95% CI, 2.48–2.83), respectively. Black women who reported high discrimination showed higher EBV VCA IgG antibody titers than Black women who reported low discrimination in the first (*p* = 0.03) and second trimesters (*p* = 0.04) as well as postpartum (*p* = 0.06). Black women who reported high discrimination showed higher EBV VCA IgG antibody titers than White women in all trimesters and postpartum (*p* < 0.001)	Moderate
Collins et al. (2000) [35]	Chicago, IL, USA	Black women (N = 85). Mothers of very-low-birth-weight (VLBW) children (*n* = 25) vs. mothers of healthy-weight children (*n* = 60).	Case-control study.	Pervasiveness of racism during pregnancy (Experiences of Discrimination Scale).	Age Education Marital status Parity Prenatal care Smoking Alcohol use Illicit drug use Social support Internalization of discrimination.	Birth weight.	The odds of giving birth to VLBW children for women who reported racial discrimination vs. those who did not was OR = 4.4 (95% CI, 1.1–18) for mothers with two or more of the following risk factors: high parity, poor pre-natal care, lacking social support, tobacco use, alcohol, or illicit drug use.	Moderate
Collins et al. (2004) [34]	Chicago, IL, USA	Black women (N = 312). Mothers of VLBW children (*n* = 104) vs. mothers of normal-weight children (*n* = 208).	Case-control study.	Pervasiveness of lifetime/pregnancy exposure to interpersonal racial discrimination (Experiences of Discrimination Scale), Frequency of experienced interpersonal racial discrimination at place of employment in past year (Perceived Racism Scale).	Age Education Marital status Parity Prenatal care Gestational age Smoking Alcohol use.	Birth weight.	Exposure to interpersonal racial discrimination during pregnancy did not appear to impact on birth weight. Lifetime exposure to racial discrimination significantly predicted VLBW. Having experienced this type of discrimination in at least one of five domains (work, getting a job, school, getting medical care, patronizing a restaurant) increased the odds of VLBW by 1.9 (95% CI, 1.2–3.1). Being the target of discrimination in three or more domains increased the odds of VLBW to 3.2 (95% CI, 1.5–6.6). This suggests a dose-response relationship. This relationship was stronger for women who had other risk factors (alcohol use, poor pre-natal care, low SES)	Moderate
Daiely et al. (2009) [2]	Northern CA, USA	Pregnant Black women (*N* = 108)	Prospective cohort study.	Frequency of experienced general discrimination (Everyday Discrimination Scale).	Exposure to traumatic event Spirituality Social support SES Lifelong trauma Smoking Alcohol Illicit drug use Pregnancy-induced hypertension Gestational diabetes Bacterial vaginosis Anemia	Birth weight.	Overall, 86% of participants reported experiences of general discrimination, typically race-, gender-, or age-based discrimination). Women who reported discrimination due to religion were more likely than women who reported no such discrimination to have infants with lower birth weight (*t* = 2.39, *p* = 0.02).	Moderate
Daniels et al. (In press) [44]	San Francisco Bay Area, CA, USA	Black women (*N* = 173)	Cross-sectional	Frequency and pervasiveness of direct and vicarious racial discrimination in three life stages (childhood, adolescence, adulthood) (Everyday Discrimination Scale).	Parity Income Education Employment status Marital status.	Preterm birth.	For each one-unit increase in adolescent direct racial discrimination, there was a 48% increase in odds for preterm labor (OR = 1.48, 95% CI, 1.00–2.19). For each one-unit increase in childhood vicarious racial discrimination, there was a 45% increase in odds for preterm labor (OR = 1.45, 95% CI, 1.01–2.09). Adult direct or vicarious racial discrimination was not statistically associated with preterm birth.	Moderate
Dixon et al. (2012) [3]	Eastern MA, USA	Mother-infant pairs (*N* = 539; Black *n* = 294, Hispanic *n* = 127, Asian *n* = 110, Other *n* = 8) in Eastern MA.	Prospective cohort study.	Pervasiveness of lifetime discrimination across eight life domains (Experiences of Discrimination Scale).	Maternal age Pre-pregnancy BMI Smoking Breastfeeding duration Nativity Gestational weight Parity College graduate Household income Sex of child Postnatal depression (during pregnancy and 6 m postpartum).	Birth weight for gestational age Age- and sex-specific weight for 6-month-old Age- and sex-specific BMI for 3-year-old.	Approx. 33% of participants had not experienced racial discrimination. However, 33% had experienced racial discrimination in 1–2 life domains, and another 35% had experienced racial discrimination in 3 or more domains. Adjusting for all covariates, children of women who had experienced discrimination in >2 life domains had infants with lower birth weight for gestational age (*β* = −0.25, 95% CI, −0.45 to −0.04), lower weight-for-age at six months (*β* = −0.34, 95% CI, −0.65 to −0.03), and lower BMI-for-age at three years (*β* = −0.33, 95% CI, −0.66 to 0.00) than children of women who had not experienced discrimination. Children of women who had experienced discrimination in 1–2 domains were generally intermediate in size and weight, suggesting a dose-response relationship.	Strong
Dole et al. (2003) [30]	Central NC, USA	Pregnant women (*N* = 1962; Black *n* = 707, White *n* = 1134, other *n* = 121).	Prospective cohort study.	Frequency of experienced racial or gender discrimination (Experiences of Discrimination Scale).	Education Age Parity Marital status Height BMI during pregnancy % poverty index Bacterial vaginosis infection Alcohol use during pregnancy Smoking during months 1–6 of pregnancy Social support Pregnancy-related anxiety Perceived neighborhood safety Stressful life events.	Preterm labor (<37 w).	There was an increased risk of preterm birth among Black women who had experienced racial discrimination (RR = 1.4, 95% CI, 1.0–2.0). Gender discrimination was not statistically associated with preterm birth.	Strong
Dole et al. (2004) [31]	Central NC, USA	Pregnant women (*N* = 1898; Black *n* = 727, White *n* = 1174).	Prospective cohort study.	Frequency of experienced racial or gender discrimination (Experiences of Discrimination Scale).	Age, Parity, Education, Marital status, Economic status, Pre-pregnancy BMI, Diet, Alcohol use, Illicit drug use, Smoking, Pre-natal care site, Bacterial vaginosis. Perceived neighborhood safety.	Preterm birth (<37 wks).	There was an increased risk of preterm birth for Black women who had experienced higher (vs. lower) levels of racial discrimination (RR = 1.8, 95% CI, 1.1–2.9). Gender discrimination predicted spontaneous preterm birth (as opposed to induced preterm) for Black women (RR = 2.1, 95% CI, 1.0–4.3).	Strong
Dominguez et al. (2008) [4]	Los Angeles, CA, USA	Pregnant women (*N* = 124; Black *n* = 51, White *n* = 73).	Prospective, repeated-measures observational study.	Pervasiveness and frequency of direct and vicarious racial discrimination in lifetime, adulthood, adolescence, childhood (Experiences of Discrimination Scale).	21 medical risk conditions, including medical history, pregnancy history, and current pregnancy. Pregnancy weight, Age, Cohabitation with father, Employment status, Race, SES, Education, Income, Income incongruity, Pregnancy stress, Gestational age, General stress.	Birth weight.	For Black women, each unit increase in lifetime perceived racism was associated with a 39.59 g decrease in birth weight, Δ*R*^2^ = 0.02, *β* = −0.17, *p* < 0.05. Accounting for life stage racism, childhood-vicarious racism emerged as the main driver of this effect with each unit increase being associated with a 167.85 g decrease in birth weight (*β* = −0.25, *p* < 0.01). Black women were more likely to give birth to children of low birth weight than White women (280.83 g difference between Black and White babies). In a fully adjusted mediation model, childhood-vicarious racism was found to mediate this effect (Δ*R*^2^ = 0.02, *p* < 0.05; Sobel test = −1.74, *p* < 0.05, one-tailed).	Moderate
Earnshaw et al. (2013) [45]	New York, NY, USA	Pregnant Black/Latina women (*N* = 420, Black *n* = 158, Latina *n* = 262).	Prospective cohort study.	Frequency of experiences of everyday discrimination (Everyday Discrimination Scale).	Age, Race/ethnicity, Birth country, Education, Relationship status, Pregnancy history, Health behavior, Nutrition, Exercise, Depression, Gestational age, Prenatal distress.	Birth weight.	Everyday discrimination was associated with greater odds of low birth weight, OR = 2.78, *p* < 0.05. The association between everyday discrimination and birth weight was mediated by increases in depressive symptoms during pregnancy (*β* = −0.04, *p* < 0.01), such that for every one-point increase in everyday discrimination, birth weight decreased by 49 g (*β* = −49.27, *p* < 0.05).	Strong
Gillespie and Anderson (2018) [36]	OH, USA	Pregnant Black women (*N* = 96).	Prospective cohort study.	Frequency and pervasiveness of lifetime exposure to racial/ethnic discrimination (Experiences of Discrimination Scale).	Pre-pregnancy BMI, Smoking, Sleep, Stress Internalization of experienced discrimination. Plasma cortisol levels, Leukocyte glucocorticoid levels.	Gestational age at birth.	There were no effects of discrimination on gestational age at birth. There was no main effect of discrimination on maternal cortisol levels. However, compared to women in discrimination tertile 1 (no discrimination, *n* = 46), women in tertile 2 (medium discrimination), who internalized experiences of discrimination (*n* = 19) exhibited higher levels of maternal cortisol (*b* = 0.68, *p* = 0.001). Racial discrimination correlated negatively with leukocyte glucocorticoid sensitivity. Each ng/mL increase in maternal cortisol level predicted birth 0.15 days earlier among women in discrimination tertile 1 vs. tertile 2 (medium) and 3 (high, *n* = 26).	Moderate
Giurgescu et al. (2012) [37]	Chicago, IL, USA	Black mothers (*N* = 72).	Descriptive correlational comparative study.	Frequency and pervasiveness of lifetime experienced racial discrimination (Experiences of Discrimination Scale).	Age, Marital status, Education, Income, Gestational age, Medical history, Psychological distress past month, Neighborhood social disorder, Perceived neighborhood physical disorder, Perceived neighborhood crime, Objective neighborhood environment.	Preterm birth.	Participants reported low levels of racial discrimination. No statistically significant relationship between racial discrimination and gestational age at birth was found. Racial discrimination predicted psychological distress (*β* = 0.524, *p* < 0.01).	Weak
Grobman et al. (2018) [38]	Nine states, USA	Pregnant women (*N* = 9470; Black *n* = 1307, White *n* = 5721, Hispanic *n* = 1586, Asian *n* = 379, other *n* = 477)	Cross-sectional study.	Frequency and pervasiveness of lifetime exposure to racial discrimination (Experiences of Discrimination Scale).	Maternal age, BMI, Smoking, Medical co-morbidities, Stress, Anxiety, Social Support, Postnatal depression, Resilience.	Preterm birth, Hypertensive disease of pregnancy, Low birth weight.	Black women were more likely than White women to give birth preterm (12.3% vs. 8.1%, OR = 1.6, 95% CI, 1.32–1.93), have low-birth weight children (17.2% vs. 8.6%, OR = 2.20, 95% CI, 1.86–2.62), and to have hypertensive disease of pregnancy (16.7 vs. 13.4, OR = 1.3, 95% CI, 1.3–1.10). None of the predictors, other than social support, accounted for statistically significant portions of variance in the discrepancy between Black and White women’s pregnancy outcomes.	Moderate
Hilmert et al. (2014) [39]	Los Angeles and Orange counties, CA, USA	Pregnant Black women (*N* = 39).	Prospective cohort study.	Pervasiveness of direct and vicarious exposure to racial discrimination in childhood and adulthood (Experiences of Discrimination Scale).	Maternal age, BMI, Parity, SES, Income, Education, Gestational age at birth.	Birth weight, Diastolic blood pressure during pregnancy.	Adjusting for confounding variables, results indicated a marginally significant inverse relationship between exposure to any racial discrimination and birth weight (*β* = −0.27, *p* < 0.10). Direct exposure to racial discrimination in adulthood was significantly and inversely associated with birth weight (*β* = −0.26, *p* < 0.05). Change in diastolic blood pressure during pregnancy and childhood vicarious and direct racial discrimination interacted to predict lower birth weight (*β* = −0.25, Δ*R*^2^ = 0.04, *p* < 0.05; *β* = −0.22, Δ*R*^2^ = 0.03, *p* < 0.10, respectively). For Black women who had experienced childhood vicarious racial discrimination in at least two life domains, birth weight declined by 19.98 g for every 1 mm Hg increase in diastolic blood pressure (*B* = −160.65, *p* < 0.05).	Moderate
Lespinasse et al. (2004) [40]	Chicago, IL, USA	Black mothers of very-low-birth-weight infants (<1500 g) (*n* = 104) vs. healthy weight infants (approx. 2500 g) (*n* = 208).	Case-control study.	Pervasiveness of lifetime experienced racial discrimination (Experiences of Discrimination Scale).	Maternal age, Marital status, Cohabitation status, Pre-natal care, Parity, Smoking, Alcohol use, Income, Desirable/undesirable pregnancy, Companion in delivery room, Stressful life events, Social environment, Religion.	Birth weight.	Experienced racial discrimination in one or more life domains was associated with a two-fold increase in the odds of very low birth weight (OR = 1.9, 95% CI, 1.2–3.0). Experienced racial discrimination in three or more life domains was associated with a near three-fold increase in the odds of very low birth weight (OR = 2.7, 95% CI, 1.3–5.4). Lack of social support was associated with a more than three-fold increase in the odds of having a baby with very low birth weight.	Strong
Mendez et al. (2014) [52]	Philadelphia, PA, USA	Pregnant women (*N* = 3462).	Cross-sectional study.	Frequency of experienced everyday discrimination, Major discriminatory instances (y/n).	Maternal race/ethnicity, Age at time of study, Income, Education, Marital status, Smoking, Alcohol use, Parity, Housing tenure/home ownership, Years lived in the neighborhood. Residential segregation, Neighborhood redlining, Stress, Neighborhood quality.	Preterm birth (<37 w).	Black women were nearly twice as likely to give birth pre-term (14.9%) than White (7.7%) or Hispanic women (8.3%). Every day and major discrimination was not associated with pre-term birth.	Weak
Misra et al. (2010) [47]	Baltimore, MD, USA	Pregnant Black women (*N* = 832).	Hybrid retrospective and prospective cohort study.	Frequency of lifetime exposure to racism (Racism and Lifetime Experiences Scale, RALES). Frequency of daily exposure to racism (RALES Daily Life Experiences Scale). Response to racism (Racism-Related Experiences, RRE Scale).	Stress, Depression symptoms, Pregnancy locus of control, Mastery, Anxiety, Social support, Maternal age, Education, Income, Family resources, SES, Smoking, Alcohol use, Illicit drug use, Vaginal douching, Parity, Pre-natal care, Chronic diseases.	Preterm birth (<37 w).	There were no main effects of either of the three racism measures on preterm birth. A score above the median on the RALES, however, was associated with an increased risk of preterm birth in women with high (vs. low) levels of stress (HR = 1.32, 95% CI, 0.64–3.57, *p* = 0.05) and in women with high (vs. low) levels of depressive symptoms (HR = 1.55, 95% CI, 0.90–2.64, *p* = 0.08). For women with low scores on both stress and depressive symptoms, experienced racism had a slight protective effect (HR = 0.63, 95% CI, 0.36–1.08).	Strong
Mustillo et al. (2004) [41]	Birmingham, AL, Chicago, IL., Oakland, CA, Minneapolis, MN, USA	Pregnant women, Chicago, IL., Oakland, CA., Minneapolis, MN. (*N* = 352).	Prospective cohort study.	Pervasiveness of lifetime experience of racial discrimination (Experiences of Discrimination Scale).	Race/ethnicity, Smoking, Alcohol use, Depressive symptoms, Education, Age, Marital status, SES Response to unfair treatment, Income.	Preterm birth (<37 w), Birth weight.	Among Black women, 50% (*n* = 16) of those with preterm deliveries and 61% (*n* = 8) of those with low-birth-weight infants had experienced racial discrimination in at least 3 situations. The corresponding rates for White women were 5% and 0%, respectively. The odds of giving birth preterm or to low-birth-weight infants were 205% (OR = 3.05, 95% CI, 1.29–7.24) and 398% (OR = 4.98, 95% CI, 1.43–17.39) greater, respectively, for women who had experienced racial discrimination >2 life domains compared to women who had not experienced discrimination. Depressive symptoms did not mediate these relationships.	Strong
Rankin et al. (2011) [49]	Chiacgo, IL, USA	Black mothers (*N* = 277).	Case-control, repeated measures study.	Frequency of past-year and lifetime experienced public-setting racism (Perceived Racism Scale), Coping with experienced racism.	Age, Education, Marital status, Parity, Prenatal care, Income, Smoking, Alcohol use.	Preterm birth (<37 w).	Lifetime and past-year experienced racial discrimination was associated with increased odds of preterm birth, (OR = 1.5, 95% CI, 0.9–2.8; OR = 2.5, 95% CI, 1.2–5.2, respectively). In terms of passive coping behavior, there was no moderating effect on the association between experienced racial discrimination and preterm birth. In terms of active coping behavior, women who reported ‘working harder to prove perpetrator wrong’ or ‘getting violent’ had lower risk of preterm birth (*p* < 0.05).	Strong
Rosenberg et al. (2002) [53]	12 states, USA	Non-Hispanic Black mothers (N = 4966). Mothers of preterm children (*n* = 422) and normal-term children (*n* = 4544).	Case-control study.	Frequency and pervasiveness of experienced racial discrimination.	Age, Parity, Previous preterm birth, Mother born preterm, Education, Smoking, Alcohol use, Second job, Asthma, Vaginal douching, High blood pressure, Diabetes during pregnancy, BMI, Geographic area of residence, Marital status.	Preterm birth (<37 w).	Women who reported unfair treatment at work (*n* = 251) were more likely to give birth preterm (OR = 1.3, 95% CI, 1.1–1.6). Women who reported that people acted afraid of them at least once a week (*n* = 50) were more likely to give birth preterm (OR = 1.4, 95% CI, 1.0–1.9). Preterm birth was also more likely for women who had less than 12 years education (*n* = 46) and who had experienced discrimination in housing (OR = 2.4, 95% CI, 1.2–4.6), in receiving service at least once a week (OR = 3.4, 95% CI, 1.5–7.7), and in terms of being feared by others at least once a week (OR = 2.0, 95% CI, 1.0–4.1).	Strong
Scholaske et al. (2019) [54]	Germany	Non-immigrant German women (*n* = 2308) and Turkish immigrants (*n* = 217).	Longitudinal nation-wide panel study	Frequency of experienced ethnic discrimination in past 12 and 24 months.	Infant sex, Maternal age, Parity, Education, Generation status (1st vs. 2nd) (for Turkish-German cohort only). BMI, Smoking during pregnancy, Pregnancy complications (e.g., hypertension, pre-eclampsia, etc.).	Preterm birth (<37 w), Birth weight.	Preterm birth was more likely for Turkish immigrants compared to non-immigrant women (*b* = 1.29, SE = 0.38, *p* < 0.001, OR = 3.61, 95% CI, 1.76–7.79). Turkish immigrants who had experienced discrimination before birth had higher risk of preterm birth (35.42%) than those who had not experienced discrimination (11.84%) (χ^2^(1, 109) = 8.18, *p* < 0.01, OR = 4.19), with lower gestational age (*t*(87.68 = 3.29, *p* < 0.01, *d* = 0.65) and birth weight (*t*(76.60 = 2.25, *p* < 0.05, *d* = 0.45). Overall, women who had experienced discrimination vs. those who had not, had a five-fold increase in risk of preterm birth, OR = 5.76, 95% CI, 1.95–19.38).	Moderate
Shiono et al. (1997) [55]	Chicago, IL, and New York, NY, USA	Pregnant Black, Chinese, Dominican, Puerto Rican, Mexican, and White women (*N* = 1150).	Cross-sectional study.	Frequency of experienced racial discrimination during pregnancy.	Maternal age, Marital status, Education, Residence, Ethnicity, Place of birth, Language, Parity, Previous abortion, Previous low-birth weight baby, Pre-pregnancy BMI, SES Insurance, Medical care, Diet Housing, Housing density, Housing stability, Anxiety, Depression, Undesired pregnancy, Locus of control, Adverse life events, Social support, Support group, Smoking, Second-hand smoking, Alcohol use, Illicit drug use, Abuse, Fasting, Exercise, Material hardship, Social adversity.	Birth weight.	In unadjusted as well as adjusted models, experienced discrimination was not associated with infant birth weight.	Weak
Slaughter-Acey et al. (2016) [50]	Detroit, MI, USA	Black women (*N* = 1410).	Retrospective cohort study.	Frequency and stressfulness of past-year experienced racial microaggressions (Daily Life Experiences of Racism and Bother (DLE-B) scale).	Prenatal depressive symptoms (1 week), Stress past month, Maternal age, Education, Financial situation, Pre-natal smoking, Self-rated physical health.	Preterm birth (<37 w).	Women in the second quartile of experienced discrimination exhibited a greater probability of preterm birth than women with higher (3rd & 4th quartile) or lower scores (1st quartile). E.g., women in the second discrimination quartile had 67% higher odds of preterm birth than women in the first discrimination quartile (OR = 1.67, 95% CI, 1.16–2.40). Women in the 3rd and 4th discrimination quartile also had greater odds of preterm birth than women in the 1st quartile, but these results were statistically non-significant. Experienced racism interacted with depression to impact preterm birth, but in a non-linear fashion. For women with mild to moderate depressive symptoms, the predictive probability of preterm birth increased from 0.10 for women in the 1st discrimination quartile (low) to 0.20 for women in the 2nd quartile (low-medium) and then decreased again to 0.10 in the 4th quartile (*p* < 0.05).	Strong
Slaughter-Acey et al. (2019) [48]	Baltimore, MD, USA	Black women (*N* = 778).	Hybrid retrospective and prospective cohort study.	Frequency of past-year and lifetime experienced racial discrimination (Racism and Life Experiences Scale). Frequency of past-year and lifetime group-based experiences of racism (Racism and Life Experiences Scale).	Parity, Recruitment status (prenatal, postnatal), Education, Employment during pregnancy, Receipts of Medicaid insurance, Maternal height, Family Resource Scale (time and money).	Birth weight for gestational age.	Maternal age and experienced racial discrimination interacted to impact on birth weight. Women over 25 (*n* = 257) had greater odds of giving birth to small-for-gestational-age babies for each one-unit increase in experienced overall and group-based racism (OR = 1.45, 95% CI, 1.02–2.08; OR = 2.84, 95% CI, 1.10–7.32, respectively). No such relationship was evident for younger women.	Strong
Thayer et al. (2019) [7]	Aotearoa, New Zealand	Pregnant Māori (*n* = 510), Pacific (*n* = 452), Asian (*n* = 691) women (N = 1653).	Longitudinal cohort study.	Pervasiveness of past-year and lifetime ethnic discrimination.	Maternal age, Maternal BMI, Household income, Education, Relationship status, Smoking, Offspring sex	Gestational age at birth, Birth weight.	Māori women who reported lifetime experiences of discrimination at work or in acquiring housing had lower-birth-weight children than those who had not experienced such discrimination (*β* = −243 g, 95% CI, −425 g, −60.2 g; (*β* = −146 g, 95% CI, −286 g, −6 g, respectively). Compared to Asian women who reported no discrimination in housing, Asian women who had experienced lifetime discrimination in housing were more likely to have higher birth-weight-children (*β* = 188 g, 95% CI, 7 g, 369 g). Shorter gestation length was evident for Māori women who reported lifetime experiences of ethnically motivated physical attacks (*β* = −1.06 week, 95% CI, −1.8 week, −0.3 week) or unfair treatment in the workplace (*β* = −0.95 week, 95% CI, −1.6 week, −0.3 week), the criminal justice system (*β* = −0.55 week, 95% CI, −1.1 week, 0.02 week), or in banking (*β* = −0.73 week, 95% CI, −1.4 week, −0.02 week).	Strong
Thayer and Kuzawa (2015) [46]	Auckland, New Zealand	Pregnant women (*N* = 64).	Prospective cohort study.	Frequency of lifetime experienced discrimination (Everyday Discrimination Scale).	Maternal age, Maternal height, Maternal weight SES Ethnicity, Education, Smoking, Exercise, Depression, Material deprivation PTSD.	Morning/evening cortisol levels, Infant stress reactivity (cortisol levels post vaccination).	Women who had been treated with less respect because of their ethnicity (*n* = 10) were more likely to self-report poor health than women who had not experienced discrimination (OR = 1.58, SE = 0.72, *p* = 0.03, *R*^2^ = 0.06). Women who had experienced ethnic discrimination exhibited (*n* = 22) higher levels of evening cortisol levels (1.25 ng/mL) than women who had experienced low levels of discrimination (0.9 ng/mL, *p* < 0.01) or none at all (0.8 ng/mL, *p* < 0.001). Infants (*n* = 19) of women who had experienced discrimination vs. those who had not, had greater cortisol response to vaccination (*β* = 6.43, SE = 2.60, *t* = 2.47, *p* < 0.05).	Moderate

## Data Availability

No new data were created or analyzed in this study. Data sharing is not applicable to this article.

## Supplementary Materials

The following are available online at https://www.mdpi.com/1660-4601/18/4/1465/s1, Table S1: PRISMA Checklist 2009. Table S2: QATQS assessment scores.

## References

[1] Allen A.M., Thomas M.D., Michaels E.K., Reeves A.N., Okoye U., Price M.M., Hasson R.E., Syme S.L., Chae D.H. (2019). Racial discrimination, educational attainment, and biological dysregulation among midlife African American women. Psychoneuroendocrinology.

[2] Dailey D.E. (2009). Social stressors and strengths as predictors of infant birth weight in low-income African American women. Nurs. Res..

[3] Dixon B., Rifas-Shiman S.L., James-Todd T., Ertel K., Krieger N., Kleinman K.P., Rich-Edwards J.W., Gillman M.W., Taveras E.M. (2012). Maternal experiences of racial discrimination and child weight status in the first 3 years of life. J. Dev. Orig. Health Dis..

[4] Dominguez T.P., Dunkel-Schetter C., Glynn L.M., Hobel C., Sandman C.A. (2008). Racial differences in birth outcomes: The role of general, pregnancy, and racism stress. Health Psychol..

[5] Olson D.M., Severson E.M., Verstraeten B.S., Ng J.W., McCreary J.K., Metz G.A. (2015). Allostatic load and preterm birth. Int. J. Mol. Sci..

[6] Pascoe E.A., Smart Richman L. (2009). Perceived discrimination and health: A meta-analytic review. Psychol. Bull..

[7] Thayer Z., Bécares L., Carr P.A. (2019). Maternal experiences of ethnic discrimination and subsequent birth outcomes in Aotearoa New Zealand. BMC Public Health.

[8] Shah P.S., Zao J., Al-Wassia H., Shah V. (2011). Pregnancy and neonatal outcomes of aboriginal women: A systematic review and meta-analysis. Women’s Health Issues.

[9] UNICEF Low Birthweight-A Good Life Starts in the Womb. https://datauniceforg/topic/nutrition/low-birthweight/.

[10] WHO Preterm Birth. https://www.who.int/news-room/fact-sheets/detail/preterm-birth.

[11] Chawanpaiboon S., Vogel J.P., Moller A.-B., Lumbiganon P., Petzold M., Hogan D., Landoulsi S., Jampathong N., Kongwattanakul K., Laopaiboon M. (2019). Global, regional, and national estimates of levels of preterm birth in 2014: A systematic review and modelling analysis. Lancet Glob. Health.

[12] Shapiro-Mendoza C.K., Barfield W.D., Henderson Z., James A., Howse J.L., Iskander J., Thorpe P.G. (2016). CDC grand rounds: Public health strategies to prevent preterm birth. Morb. Mortal. Wkly. Rep..

[13] Giurgescu C., McFarlin B.L., Lomax J., Craddock C., Albrecht A. (2011). Racial discrimination and the Black-White gap in adverse birth outcomes: A review. J. Midwifery Women’s Health.

[14] Mantell C.D., Craig E.D., Stewart A.W., Ekeroma A.J., Mitchell E.A. (2004). Ethnicity and birth outcome: New Zealand trends 1980–2001: Part 2. Pregnancy outcomes for Maori women. Aust. N. Z. J. Obstet. Gynaecol..

[15] Blumenshine P., Egerter S., Barclay C.J., Cubbin C., Braveman P.A. (2010). Socioeconomic disparities in adverse birth outcomes: A systematic review. Am. J. Prev. Med..

[16] CDC Preterm Birth. https://www.cdc.gov/reproductivehealth/maternalinfanthealth/pretermbirth.htm.

[17] Rich-Edwards J.W., Grizzard T.A. (2005). Psychosocial stress and neuroendocrine mechanisms in preterm delivery. Am. J. Obstet. Gynecol..

[18] McEwen B.S. (2000). Allostasis and allostatic load: Implications for neuropsychopharmacology. Neuropsychopharmacology.

[19] Beckie T.M. (2012). A systematic review of allostatic load, health, and health disparities. Biol. Res. Nurs..

[20] Black P.H., Garbutt L.D. (2002). Stress, inflammation and cardiovascular disease. J. Psychosom. Res..

[21] McEwen B.S., Stellar E. (1993). Stress and the individual: Mechanisms leading to disease. Arch. Intern. Med..

[22] Hux V.J., Catov J.M., Roberts J.M. (2014). Allostatic load in women with a history of low birth weight infants: The national health and nutrition examination survey. J. Women’s Health.

[23] Hux V.J., Roberts J.M. (2015). A potential role for allostatic load in preeclampsia. Matern. Child Health J..

[24] Dominguez T.P. (2011). Adverse birth outcomes in African American women: The social context of persistent reproductive disadvantage. Soc. Work Public Health.

[25] Giscombé C.L., Lobel M. (2005). Explaining disproportionately high rates of adverse birth outcomes among African Americans: The impact of stress, racism, and related factors in pregnancy. Psychol. Bull..

[26] Giurgescu C., Misra D.P. (2018). Psychosocial factors and preterm birth among black mothers and fathers. MCN Am. J. Matern. Child Nurs..

[27] Alhusen J.L., Bower K.M., Epstein E., Sharps P. (2016). Racial discrimination and adverse birth outcomes: An integrative review. J. Midwifery Women’s Health.

[28] Mutambudzi M., Meyer J.D., Reisine S., Warren N. (2017). A review of recent literature on materialist and psychosocial models for racial and ethnic disparities in birth outcomes in the US, 2000–2014. Ethn. Health.

[29] Thomas B., Ciliska D., Dobbins M., Micucci S. (2004). A process for systematically reviewing the literature: Providing the research evidence for public health nursing interventions. Worldviews Evid.-Based Nurs..

[30] Dole N., Savitz D.A., Hertz-Picciotto I., Siega-Riz A.M., McMahon M.J., Buekens P. (2003). Maternal stress and preterm birth. Am. J. Epidemiol..

[31] Dole N., Savitz D.A., Siega-Riz A.M., Hertz-Picciotto I., McMahon M.J., Buekens P. (2004). Psychosocial factors and preterm birth among African American and White women in central North Carolina. Am. J. Public Health.

[32] Krieger N., Smith K., Naishadham D., Hartman C., Barbeau E.M. (2005). Experiences of discrimination: Validity and reliability of a self-report measure for population health research on racism and health. Soc. Sci. Med..

[33] Christian L.M., Iams J.D., Porter K., Glaser R. (2012). Epstein-Barr virus reactivation during pregnancy and postpartum: Effects of race and racial discrimination. Brain Behav. Immun..

[34] Collins J.W., David R.J., Handler A., Wall S., Andes S. (2004). Very low birthweight in African American infants: The role of maternal exposure to interpersonal racial discrimination. Am. J. Public Health.

[35] Collins J.W., David R.J., Symons R., Handler A., Wall S.N., Dwyer L. (2000). Low-income African-American mothers’ perception of exposure to racial discrimination and infant birth weight. Epidemiology.

[36] Gillespie S.L., Anderson C.M. (2018). Racial discrimination and leukocyte glucocorticoid sensitivity: Implications for birth timing. Soc. Sci. Med..

[37] Giurgescu C., Zenk S.N., Dancy B.L., Park C.G., Dieber W., Block R. (2012). Relationships among neighborhood environment, racial discrimination, psychological distress, and preterm birth in African American women. J. Obstet. Gynecol. Neonatal Nurs..

[38] Grobman W.A., Parker C.B., Willinger M., Wing D.A., Silver R.M., Wapner R.J., Simhan H.N., Parry S., Mercer B.M., Haas D.M. (2018). Racial disparities in adverse pregnancy outcomes and psychosocial stress. Obstet. Gynecol.

[39] Hilmert C.J., Dominguez T.P., Schetter C.D., Srinivas S.K., Glynn L.M., Hobel C.J., Sandman C.A. (2014). Lifetime racism and blood pressure changes during pregnancy: Implications for fetal growth. Health Psychol..

[40] Lespinasse A.A., David R.J., Collins J.W., Handler A.S., Wall S.N. (2004). Maternal support in the delivery room and birthweight among African-American women. J. Natl. Med. Assoc..

[41] Mustillo S., Krieger N., Gunderson E.P., Sidney S., McCreath H., Kiefe C.I. (2004). Self-reported experiences of racial discrimination and Black–White differences in preterm and low-birthweight deliveries: The CARDIA Study. Am. J. Public Health.

[42] Williams D.R., Yu Y., Jackson J.S., Anderson N.B. (1997). Racial differences in physical and mental health: Socio-economic status, stress and discrimination. J. Health Psychol..

[43] Carty D.C., Kruger D.J., Turner T.M., Campbell B., DeLoney E.H., Lewis E.Y. (2011). Racism, health status, and birth outcomes: Results of a participatory community-based intervention and health survey. J. Urban Health.

[44] Daniels K.P., Zulema V., Chae D.H., Allen A.M. (2020). Direct and vicarious racial discrimination at three life stages and preterm labor: Results from the African American Women’s Heart & Health Study. Matern. Child Health J..

[45] Earnshaw V.A., Rosenthal L., Lewis J.B., Stasko E.C., Tobin J.N., Lewis T.T., Reid A.E., Ickovics J.R. (2013). Maternal experiences with everyday discrimination and infant birth weight: A test of mediators and moderators among young, urban women of color. Ann. Behav. Med..

[46] Thayer Z.M., Kuzawa C.W. (2015). Ethnic discrimination predicts poor self-rated health and cortisol in pregnancy: Insights from New Zealand. Soc. Sci. Med..

[47] Misra D., Strobino D., Trabert B. (2010). Effects of social and psychosocial factors on risk of preterm birth in black women. Paediatr. Perinat. Epidemiol..

[48] Slaughter-Acey J.C., Talley L.M., Stevenson H.C., Misra D.P. (2019). Personal versus group experiences of racism and risk of delivering a small-for-gestational age infant in African American women: A life course perspective. J. Urban Health.

[49] Rankin K.M., David R.J., Collins J.W. (2011). African American women’s exposure to interpersonal racial discrimination in public settings and preterm birth: The effect of coping behaviors. Ethn. Dis..

[50] Slaughter-Acey J.C., Sealy-Jefferson S., Helmkamp L., Caldwell C.H., Osypuk T.L., Platt R.W., Straughen J.K., Dailey-Okezie R.K., Abeysekara P., Misra D.P. (2016). Racism in the form of micro aggressions and the risk of preterm birth among black women. Ann. Epidemiol..

[51] Bower K.M., Geller R.J., Perrin N.A., Alhusen J. (2018). Experiences of racism and preterm birth: Findings from a pregnancy risk assessment monitoring system, 2004 through 2012. Women’s Health Issues.

[52] Mendez D.D., Hogan V.K., Culhane J.F. (2014). Institutional racism, neighborhood factors, stress, and preterm birth. Ethn. Health.

[53] Rosenberg L., Palmer J.R., Wise L.A., Horton N.J., Corwin M.J. (2002). Perceptions of racial discrimination and the risk of preterm birth. Epidemiology.

[54] Scholaske L., Brose A., Spallek J., Entringer S. (2019). Perceived discrimination and risk of preterm birth among Turkish immigrant women in Germany. Soc. Sci. Med..

[55] Shiono P.H., Rauh V.A., Park M., Lederman S.A., Zuskar D. (1997). Ethnic differences in birthweight: The role of lifestyle and other factors. Am. J. Public Health.

[56] Geronimus A.T. (1992). The weathering hypothesis and the health of African-American women and infants: Evidence and speculations. Ethn. Dis..

[57] Haslam C., Jetten J., Cruwys T., Dingle G., Haslam S.A. (2018). The New Psychology of Health: Unlocking the Social Cure.

[58] Major B., Dovidio J.F., Link B.G. (2018). The Oxford Handbook of Stigma, Discrimination, and Health.

[59] Brody G.H., Lei M.K., Chae D.H., Yu T., Kogan S.M., Beach S.R. (2014). Perceived discrimination among African American adolescents and allostatic load: A longitudinal analysis with buffering effects. Child Dev..

[60] Chae D.H., Lincoln K.D., Adler N.E., Syme S.L. (2010). Do experiences of racial discrimination predict cardiovascular disease among African American men? The moderating role of internalized negative racial group attitudes. Soc. Sci. Med..

[61] Forsyth J., Schoenthaler A., Chaplin W.F., Ogedegbe G., Ravenell J. (2014). Perceived discrimination and medication adherence in black hypertensive patients: The role of stress and depression. Psychosom. Med..

[62] Geronimus A.T., Hicken M., Keene D., Bound J. (2006). “Weathering” and age patterns of allostatic load scores among blacks and whites in the United States. Am. J. Public Health.

[63] Guyll M., Matthews K.A., Bromberger J.T. (2001). Discrimination and unfair treatment: Relationship to cardiovascular reactivity among African American and European American women. Health Psychol..

[64] Mays V.M., Cochran S.D., Barnes N.W. (2007). Race, race-based discrimination, and health outcomes among African Americans. Annu. Rev. Psychol..

[65] Nuru-Jeter A., Dominguez T.P., Hammond W.P., Leu J., Skaff M., Egerter S., Jones C.P., Braveman P. (2009). “It’s the skin you’re in”: African-American women talk about their experiences of racism. An exploratory study to develop measures of racism for birth outcome studies. Matern. Child Health J..

[66] Braveman P., Heck K., Egerter S., Dominguez T.P., Rinki C., Marchi K.S., Curtis M. (2017). Worry about racial discrimination: A missing piece of the puzzle of Black-White disparities in preterm birth?. PLoS ONE.

[67] Brown-Iannuzzi J.L., Hoffman K.M., Payne B.K., Trawalter S. (2014). The invisible man: Interpersonal goals moderate inattentional blindness to African Americans. J. Exp. Psychol. Gen..

[68] Franklin A.J., Boyd-Franklin N. (2000). Invisibility syndrome: A clinical model of the effects of racism on African-American males. Am. J. Orthopsychiatry.

[69] Smart Richman L., Leary M.R. (2009). Reactions to discrimination, stigmatization, ostracism, and other forms of interpersonal rejection: A multimotive model. Psychol. Rev..

[70] Torres-Harding S., Turner T. (2015). Assessing racial microaggression distress in a diverse sample. Eval. Health Prof..

[71] Sutton A.J. (2009). Publication bias. The Handbook of Research Synthesis and Meta-Analysis.

[72] Simons R.L., Lei M., Beach S.R.H., Barr A.B., Simons L.G., Gibbons F.X., Philibert R.A. (2018). Discrimination, segregation, and chronic inflammation: Testing the weathering explanation for the poor health of Black Americans. Dev. Psychol..

[73] Schmeer K.K., Tarrence J. (2018). Racial-ethnic disparities in inflammation: Evidence of weathering in childhood?. J. Health Soc. Behav..

[74] Forde A.T., Crookes D.M., Suglia S.F., Demmer R.T. (2019). The weathering hypothesis as an explanation for racial disparities in health: A systematic review. Ann. Epidemiol..

[75] Turner R.N., West K. (2012). Behavioural consequences of imagining intergroup contact with stigmatized outgroups. Group Process. Intergroup Relat..

[76] Bailey Z.D., Krieger N., Agénor M., Graves J., Linos N., Bassett M.T. (2017). Structural racism and health inequities in the USA: Evidence and interventions. Lancet.

[77] Visionary Vanguard Group Inc (2017). The JJ Way^®^: Community-Based Maternity Center, Final Evaluation Report.

[78] Larrabee Sonderlund A., Thilsing T., Sondergaard J. (2019). Should social disconnectedness be included in primary-care screening for cardiometabolic disease? A systematic review of the relationship between everyday stress, social connectedness, and allostatic load. PLoS ONE.

