# Supplementary material for: The Association between Maternal Experiences of Interpersonal Discrimination and Adverse Birth Outcomes: A Systematic Review of the Evidence
Source: Int J Environ Res Public Health. 2021 Feb 4;18(4):1465. doi: 10.3390/ijerph18041465 (PMC7913961; doi:10.3390/ijerph18041465)
Supplement: Supplementary file 1 [file ijerph-18-01465-s001.zip › S2 QAQTS assessment scores.docx]

S2 Quality assessment scores

| **Study** | **Selection bias** | **Design** | **Confounders** | **Blinding** | **Data collection methods** | **Withdrawals** | **Overall score** |
| --- | --- | --- | --- | --- | --- | --- | --- |
| Bower et al. (2018) | Moderate | Weak | Moderate | Moderate | Moderate | Moderate | Moderate |
| Carty et al. (2011) | Moderate | Weak | Moderate | Moderate | Strong | Moderate | Moderate |
| Christian et al. (2012) | Weak | Moderate | Moderate | Moderate | Strong | Moderate | Moderate |
| Collins et al. (2000) | Weak | Moderate | Moderate | Moderate | Strong | Moderate | Moderate |
| Collins et al. (2004) | Moderate | Moderate | Weak | Moderate | Strong | Moderate | Moderate |
| Daiely et al. (2009) | Weak | Moderate | Moderate | Moderate | Strong | Moderate | Moderate |
| Daniels et al. (In press) | Moderate | Moderate | Weak | Moderate | Strong | Moderate | Moderate |
| Dixon et al. (2012) | Moderate | Moderate | Moderate | Moderate | Strong | Moderate | Strong |
| Dole et al. (2003) | Moderate | Moderate | Strong | Moderate | Strong | Moderate | Strong |
| Dole et al. (2004) | Moderate | Moderate | Strong | Moderate | Strong | Moderate | Strong |
| Dominguez et al. (2008) | Weak | Moderate | Strong | Moderate | Strong | Moderate | Moderate |
| Earnshaw et al. (2013) | Moderate | Moderate | Moderate | Moderate | Strong | Moderate | Strong |
| Gillespie & Anderson (2018) | Weak | Moderate | Moderate | Moderate | Strong | Moderate | Moderate |
| Giurgescu et al. (2012) | Weak | Weak | Moderate | Moderate | Strong | Moderate | Weak |
| Grobman et al. (2018) | Moderate | Weak | Moderate | Moderate | Strong | Moderate | Moderate |
| Hilmert et al. (2014) | Weak | Moderate | Moderate | Moderate | Strong | Moderate | Moderate |
| Lespinasse et al. (2004) | Moderate | Moderate | Strong | Moderate | Strong | Moderate | Strong |
| Mendez et al. (2014) | Moderate | Weak | Moderate | Moderate | Weak | Moderate | Weak |
| Misra et al. (2010) | Moderate | Moderate | Strong | Moderate | Strong | Moderate | Strong |
| Mustillo et al. (2004) | Moderate | Moderate | Moderate | Moderate | Strong | Moderate | Strong |
| Rankin et al. (2011) | Moderate | Moderate | Moderate | Moderate | Strong | Moderate | Strong |
| Rosenberg et al. (2002) | Moderate | Moderate | Strong | Moderate | Moderate | Moderate | Strong |
| Scholaske et al. (2019) | Moderate | Moderate | Moderate | Moderate | Weak | Moderate | Moderate |
| Shiono et al. (1997) | Moderate | Weak | Moderate | Moderate | Weak | Moderate | Weak |
| Slaughter-Acey et al. (2016) | Moderate | Moderate | Moderate | Moderate | Strong | Moderate | Strong |
| Slaughter-Acey et al. (2019) | Moderate | Moderate | Moderate | Moderate | Strong | Moderate | Strong |
| Thayer et al. (2019) | Moderate | Moderate | Moderate | Moderate | Moderate | Moderate | Strong |
| Thayer & Kuzawa (2015) | Weak | Moderate | Moderate | Moderate | Strong | Moderate | Moderate |
